# Exploration of the Fusidic Acid Structure Activity Space for Antibiotic Activity

**DOI:** 10.3390/molecules30030465

**Published:** 2025-01-21

**Authors:** Yoon-Suk Kang, Simone C. Silva, Kenneth Smith, Krissty Sumida, Yuhan Wang, Lucius Chiaraviglio, Ramachandra Reddy Donthiri, Alhanouf Z. Aljahdali, James E. Kirby, George A. O’Doherty

**Affiliations:** 1 Department of Pathology, Beth Israel Deaconess Medical Center, Boston, MA 02115, USA; ykang3@bidmc.harvard.edu (Y.-S.K.); kpsmith2201@gmail.com (K.S.); 2 Harvard Medical School, Boston, MA 02115, USA; 3 Department of Chemistry, Northeastern University, Boston, MA 02115, USA; mone_sii@yahoo.com.br (S.C.S.); ksumida16@gmail.com (K.S.); wang.yuhan8@northeastern.edu (Y.W.)

**Keywords:** fusidic acid, structure–activity relationship, antibiotic natural product antibiotic

## Abstract

Fusidic acid is a translation inhibitor with activity against major Gram-positive bacterial pathogens such as *S. aureus*. However, its activity against Gram-negatives is poor based on an inability to access its cytoplasmic target in these organisms. Opportunities for functionalization of the fusidic acid scaffold to enhance activity against Gram-negative pathogens have not been explored. Using an activity-guided synthetic strategy, the tolerance of the tetracyclic natural product to derivatization at the A- and C-rings and its carboxylic acid side chain was explored with the goal of enhancing its activity spectrum and pharmacological properties. All side-chain carboxylic acid esters were inactive. Oxidation of the C-ring alcohol and oxime were not tolerated either. A number of esters of the A-ring alcohol retained modest activity against Gram-positive bacteria and were informative for future activity-guided studies. For the A-ring esters, differences in antibacterial activity relative to inhibitory activity in a ribosome in vitro translation assay suggested the possibility of a pro-druglike effect for the fusidic acid pyrazine-2-carboxylate. This study furthers the understanding of the activity of the fusidic acid scaffold against Gram-positive bacteria. These results suggest promise for future modification of the A-ring alcohol of fusidic acid in the advancement of its antibiotic properties.

## 1. Introduction

Fusidic acid is a steroid antimicrobial natural product with potent activity against methicillin-resistant *Staphylococcus aureus* (MRSA) and methicillin-susceptible *Staphylococcus aureus* (MSSA), β-hemolytic streptococci; potent in vitro activity against *Neisseria gonorrhoeae*; and in vitro and in vivo activity against extensively drug-resistant *Mycobacterium tuberculosis* [1,2,3]. There is clearly a great need for new Gram-negative antibiotics that can reduce selective resistance pressure through diversification of our antimicrobial therapeutic portfolio [4]. Our approach to the problem of antibiotic resistance centers on the derivatization of existing antibiotic natural products [5,6]. The drug is water-soluble, stable (shelf life 3 years), and highly orally available, and therefore can be given intravenously, orally, or used topically [7]. It has an excellent safety profile [8,9], a long half-life in humans, and is eliminated through biliary excretion. Fusidic acid concentrates well in all tissues; however, it does not cross the blood–brain barrier.

Fusidic acid targets ribosome translocation by inhibiting function of the ribosome-associated protein elongation factor G (EF-G), which assists in the translocation of transfer RNA in the ribosome. It achieved primary endpoints in a phase III US clinical trial (i.e., non-inferiority to linezolid in acute bacterial skin and skin structure infections) [1]. It is generally considered a static agent, but killing of *Staphylococcus aureus* and especially *Streptococcus pyogenes* is evident in hollow-fiber infection models [10]. Resistance to fusidic acid has been found in *S. aureus* [11] as a result of single-step mutations in fusidic acid’s ribosomal target, EF-G (encoded by the gene *fusA*), conferring high-level resistance, and in the ribosomal protein RplF (encoded by the gene *fusE*). Although multiple mutations can be isolated in the laboratory, only a minority (e.g., organisms encoded with *fusA*, L461K, and to a lesser extent H457Y and H457Q) are observed in clinical isolates and at low frequency, suggesting a significant fitness cost, supported by in vitro and in vivo data, including human colonization experiments [12,13,14,15]. Higher loading dose regimens have further addressed concern about selection of *fusA* resistance during monotherapy [1]. Plasmid-mediated, low-level resistance conferred by the FusB family proteins are the predominant resistance mechanism in Gram-positive clinical isolates. These proteins bind to EF-G and overcome the inhibitory effect of fusidic acid [16,17,18].

Brennan-Krohn et al. recently described fusidic acid’s potent and broad-spectrum activity against highly resistant, carbapenemase-producing *Enterobacterales* (CRE) when combined with colistin, which is known to permeabilize the outer Gram-negative bacterial membrane [19]. This co-dosing effect proved to be true even in colistin-resistant strains. We further note its potent activity against intracellular *Legionella pneumophila* [20] and facultative intracellular *Brucella neotomae* (unpublished). Others have also identified fusidic acid as having compelling axenic activity against *L pneumophila* when grown axenically [21,22]. These fastidious organisms have a lower permeation barrier to prototypical Gram-positive agents. Potent activity is also noted in *Escherichia coli* (*E. coli*) mutants with increased permeation (i.e., *ΔtolC* or *lptD4213* genetic mutations) [23,24]. Notably, polymyxin B nonapeptide, an outer membrane permeabilization agent without direct antimicrobial activity, confers drastically reduced fusidic acid MICs for *Klebsiella pneumonia*, *Acinetobacter baumannii*, and *Pseudomonas aeruginosa* into the 1 μg/mL range [25]. Notably, FusB, and presumably related FusB family proteins, do not bind to *E. coli* EF-G and fail to overcome fusidic acid inhibition of *E. coli* in vitro translation systems [26,27]. Taken together, these results suggest that fusidic acid would have potent Gram-negative activity if it could penetrate the outer Gram-negative membrane, as there are no known pre-existing resistance mechanisms circulating in these pathogens [28].

**Figure 1:**
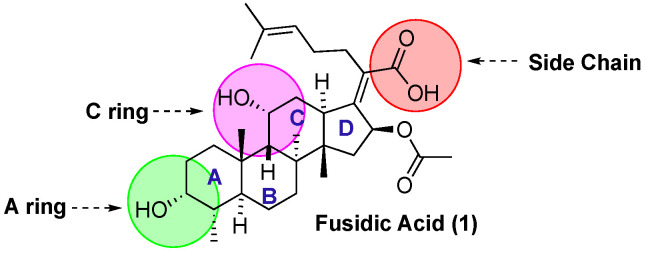
Fusidic acid structure and functionalization sites.

As part of a long-standing interest in natural product medicinal chemistry, we became interested in the structural space around fusidic acid [29,30,31,32,33]. In particular, we wanted to explore whether the antibacterial activity of fusidic acid could be enhanced through derivatization. The fusidic acid scaffold has been previously investigated by others [34,35], most notably investigating the anti-malarial structure–activity relationship (SAR) [36,37]. A series of analogues modified at C-21 showed improved potency against *P. falciparum* [38]. Other have also explored modification of the A- and C-ring alcohols (Figure 1) [39,40,41].

## 2. Results and Discussion

Our own SAR efforts were aimed at identifying sites that showed promise of systematic variation compatible with generation and screening of large libraries of compounds. Specifically, we looked to identify which portions of the tetracyclic steroidal core of fusidic acid were amenable to functionalization without disrupting its potency as an antibacterial agent. The knowledge gained from this effort would in turn aid future studies aimed at expanding its antimicrobial activity spectrum. Initial efforts aimed at the de novo design using molecular modeling/docking studies of potential analogues was limited by the difficulty in finding a static structure that replicated the known X-ray and EPR structures of fusidic acid bound to EF-G–ribosome complexes [39,40,41,42,43,44]. As a result, we settled on the use of an activity-guided synthetic/screening strategy. For the screening, we chose to use *S. aureus* as the Gram-positive model organism and three types of *E. coli*: a wild type (*E. coli* wt), an *E. coli* with its TolC efflux transporter protein knocked out (*E. coli ΔtolC*) [45], and an *E. coli* mutant with a hyperpermeable outer membrane due to defective lipopolysaccharide transport (*E. coli LptD4213*) [46]. An *E. coli*-based in vitro transcription-translation assay was also used to validate that any of the newly discovered analogues with anti-*S. aureus* or anti-*E. coli* activity were targeting the ribosome [24]. At the outset, we identified three sites on the molecule that could be easily derivatized (Figure 1): the side-chain carboxylic acid (esters), the A ring C-3 alcohol (ketone, oxime and esters), and the C ring C-11 alcohol (ketone and oxime). Herein, we report the results of this effort.

**Scheme 1:**
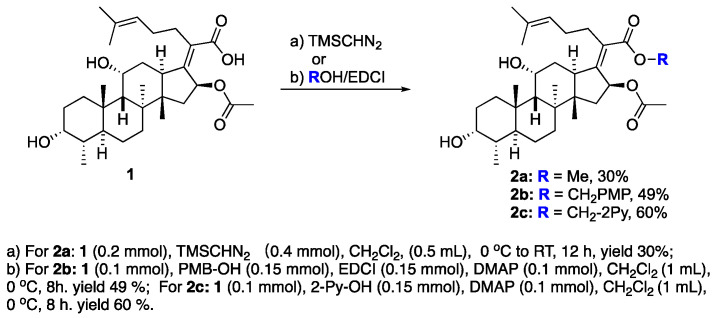
Synthesis of side-chain ester analogues of fusidic acid.

The preparation of side-chain ester analogues began with the straightforward conversion of fusidic acid (**1**) into carboxylic acid esters. Specifically, we chose the smallest possible methyl ester, **2a**, as well as the larger electronic rich para-methoxybenzyl ester **2b** and electron-deficient 2-pyridylmethylester **2c** with a basic nitrogen (Scheme 1). The known methyl ester **2a** was easily prepared upon treatment with excess TMS-diazomethane in CH_2_Cl_2_, affording **2a** with a 30% yield. In this case, the diol functionality in fusidic acid alleviated the need for alcohol solvents [47]. The other two esters, **2b** and **2c**, were prepared using carbodiimide coupling chemistry, specifically 1-ethyl-3-(3-dimethylaminopropyl)carbodiimide (EDCI). The selectivity issues associated with the fact that the fusidic acid structure contains both a carboxylic acid and two secondary alcohols were easily addressed by performing the coupling with a slight excess of alcohol (1.5 equiv), affording **2b** and **2c** with 49% and 60% yields, respectively. Because we prioritized purity over yield, the yields for these transformations were not optimized. Three purified esters were screened for antibiotic activity against *S. aureus*, and either a wild type *E. coli* K-12 strain or *E. coli* strains with increased permeability characteristics (*ΔtolC* and *lptD4213* mutants) [48]. Unfortunately, no activity was found for the three different esters **2a**–**c** (Table 1). Interestingly, this contrasted with prior reports of somewhat better activity for methyl ester **2a** and a small subset of additional side-chain esters [41]. Therefore, we turned our efforts toward the generation of analogues that were functionalized on the A-ring and C-ring.

**Scheme 2:**
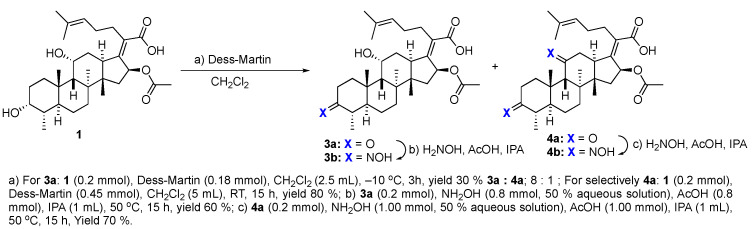
Modification of the A-ring.

We next explored the effects on antibacterial activity of A- and C-ring substitution (Scheme 2). To accomplish this, we searched for reaction chemistry that selectively reacted at the A-ring alcohol over the alcohol in the C-ring and vice versa. We first explored oxidation chemistry to selectively access a mono-ketone in the A- or C-ring. For steric reasons, it is reasonable to assume that the A-ring alcohol would be more accessible to reagent over the C-ring. To some extent, this proved to be true for the oxidation of fusidic acid with Dess–Martin periodate. That is to say, the A-ring alcohol was the more reactive, although it was difficult to prevent overoxidation to the diketone. Thus, under our optimized conditions, a 30% yield of fusidic acid mono-ketone was prepared when fusidic acid **3a** was reacted with 0.9 equivalent Dess–Martin reagent. In contrast, the further oxidized diketone **4a** product was significantly easier to obtain when excess (2.5 equiv) Dess–Martin reagent was used (80% yield). To further diversify the fusidic acid structural motifs, ketones **3a** and **4a** were converted into oximes **3b** and **4b**. This was simply accomplished with mono-ketone **3a** by exposing it to excess aqueous hydroxyamine (4 equiv). Under these conditions, the A-ring oxime **3b** was able to be isolated with a 60% yield. Under similar conditions with a slightly higher amount of aqueous hydroxyamine (5 equiv), the bisketone **4a** was able to be converted into bisoxime **4b** with a 70% yield. The four analogues **3a/b** and **4a/b** were tested for their ability to inhibit growth of *S. aureus* and *E. coli* strains and for their ability to inhibit in vitro translation in an assay performed with *E. coli* S30 ribosome extracts (Table 2). In contrast to a complete block of antimicrobial activity observed with carboxylic acid esterification, the fusidic acid A-ring oxidation retained modest antimicrobial activity against *S. aureus*. Others have found similar activity for mono-ketone **3a** and oxime **3b** against *S. aureus* and mycobacteria [45,46]. As for fusidic acid, active analogues did not have observable activity in wild-type *E. coli* or its hyperpermeable *lptD4213* mutant at the concentrations tested, but did have activity in the *ΔtolC* mutant, consistent with exclusion of fusidic acid and its analogues by efflux pump mechanisms. Activity in the *E. coli* in vitro translation assay (IC_50_) correlated with antimicrobial activity (MIC) against *S. aureus*, consistent with the ability of fusidic acid and active analogues to inhibit translation if they gain access to their ribosomal target. Unfortunately, further oxidation of the C-ring and/or oxime formation had a deleterious effect on activity.

**Scheme 3:**
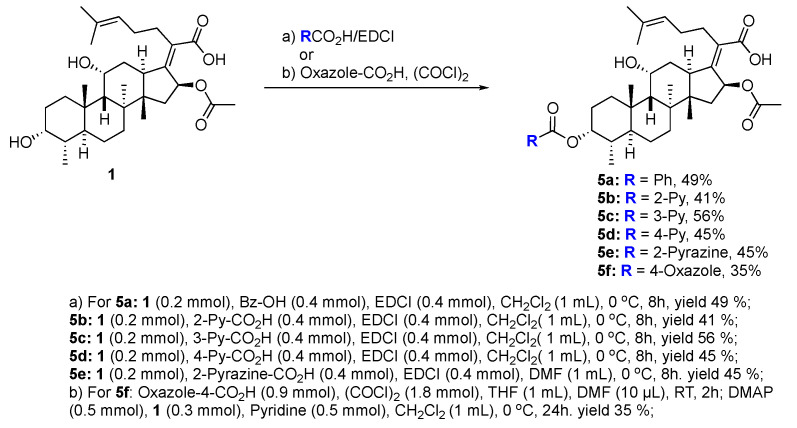
Synthesis of A-ring esters.

We next decided to further explore substitution of the fusidic acid A-ring with ester formation with a series of aromatic carboxylic acids. The structure of the aromatic ring was varied by the inclusion of one to two basic nitrogen atoms in the ortho, meta, and para portion of the benzene ring. To accomplish this, we returned to the use of carbodiimide coupling chemistry. In contrast to the condition used for the selective coupling of the fusidic acid carboxylic acid with alcohols, we chose to first premix the EDCI and the desired carboxylic acid coupling partner before exposure to fusidic acid. Typically, the acylurea intermediate that is formed from the carboxylic acid–EDCI carboxylic acid/EDCI admixture was used in a two-fold excess to fusidic acid. Under these conditions, fusidic acid A-ring esters **5a**–**e** were exclusively formed and able to be isolated, with yields ranging from 41% to 56% (Scheme 3). The six analogues **5a**–**f** were screened for antibiotic activity (see Table 3).

While mostly inactive against *E. coli*, interesting activity was observed against *S. aureus*. This was especially true for ester **5e**, the esterification product with pyrazine-2-carboxylic acid, which had an MIC of 0.5 μM. It appears both nitrogen atoms played a role in the activity of **5e**, as when one or both nitrogen atoms were removed to form either 2-pyridyl **5b** or 3-pyridyl **5c**, activity was reduced (16 μM). In addition, when the nitrogen was moved to a 1,4-relationship to the ester carboxylate, as in **5d**, the activity dropped significantly (64 μM). The oxazole analogue **5f**, which also has an SP^2^ nitrogen atom in a 1,2-relationship to the carboxylation, had similar activity (16 μM). However, it should be noted that the second-most active compound against *S. aureus* was the benzoate **5a** (2–4 μM) with both nitrogen atoms removed. Analogues **5a**–**f** were also evaluated for inhibitor effects on in vitro translation. With the exception of the 4-pyridyl analogue **5d,** all the compounds showed similar ribosome inhibitory activity. Interestingly, the analogue most active against *S. aureus*, **5e,** did not have corresponding potency in in vitro translation assays. This suggested the possibility that the improved antibacterial activity of **5e** might be due to the selective hydrolysis of the pyrazine-2-carboxylate to the more potent fusidic acid. The hydrolysis of the more reactive pyrazine-2-carboxylate ester in **5e** could have been enzyme-promoted or occurred during the longer incubation time used during antibacterial testing. However, potential improvements in ability to access the cytoplasmic EF-G target relative to intrinsic potency cannot be ruled out.

## 3. Materials and Methods

### 3.1. General Methods

^1^H and ^13^C{1H} spectra were recorded on Bruker 500 and 126 MHz NMR spectrometers, see the Supplementary Materials Section. Chemical shifts were reported relative to internal tetramethylsilane (δ 0.00 ppm) or CDCl_3_ (δ 7.26 ppm) or CD_3_OD (δ 4.89 ppm) for ^1^H-NMR and CDCl_3_ (δ 77.1 ppm) or CD_3_OD (δ 49.15 ppm) for ^13^C{1H}-NMR. Optical rotations were measured with a digital polarimeter in the solvent specified. Infrared (IR) spectra were obtained on a Bruker FT-IR spectrometer. Flash column chromatography was performed on ICN reagent 60 (60–200 mesh) silica gel. Analytical thin-layer chromatography was performed with precoated glass-backed plates (K6F 60 Å, F254) and visualized by quenching of fluorescence and charring after treatment with cerium ammonium molybdate or potassium permanganate stain. *R_f_* values were obtained by elution in the stated solvent ratios (*v*/*v*). Ether, tetrahydrofuran (THF), dimethyl formamide (DMF), methylene chloride (DCM), methanol (MeOH), ethyl acetate (EA), hexane, and triethylamine (TEA) were dried by passing through activated silica gel (8 × 14 mesh) column with nitrogen gas pressure. All commercial reagents were obtained from the Fisher Scientific (Waltham, MA, USA) used without purification unless otherwise noted. Air- and/or moisture-sensitive reactions were carried out under an atmosphere of argon/nitrogen using oven/flame-dried glassware and standard syringe/septa techniques.

### 3.2. Experimental Procedures

**methyl(*Z*)-2-((3*R*,4*S*,5*S*,8*S*,9*S*,10*S*,11*R*,13*R*,14*S*,16*S*)-16-acetoxy-3,11-dihydroxy-4,8,10,14-tetramethylhexadecahydro-17H-cyclopenta[a]phenanthren-17-ylidene)-6-methylhept-5-enoate (2a)**

A solution was prepared by dissolving fusidic acid (100 mg, 0.194 mmol, 1 equiv) in DCM (0.5 mL) and was cooled to 0 °C. A (0.2 mL) solution of TMSCH_2_N_2_, made from a (2M solution in ether) was added slowly at 0 °C. The reaction was brought up to room temperature and stirred for 12 h, then directly loaded onto a silica gel column. Flash chromatography (30% ethyl acetate in hexanes) gave **2a** (colorless solid 93 mg, 90%); *R_f_* = 0.58 (60% ethyl acetate/hexanes):

^1^H NMR (500 MHz, CDCl_3_) δ 5.84 (d, *J* = 8.4 Hz, 1H), 5.08 (tt, *J* = 7.2, 1.6 Hz, 1H), 4.34 (q, *J* = 2.7 Hz, 1H), 3.75 (q, *J* = 2.8 Hz, 1H), 3.63 (s, 3H), 3.05–3.01 (m, 1H), 2.53–2.36 (m, 2H), 2.30 (dt, *J* = 13.2, 3.3 Hz, 1H), 2.18 (dd, *J* = 13.0, 4.2 Hz, 1H), 2.11 (ddd, *J* = 16.5, 11.6, 5.4 Hz, 2H), 2.06–1.98 (m, 1H), 1.97 (s, 3H), 1.91–1.70 (m, 4H), 1.66 (d, *J* = 1.5 Hz, 3H), 1.62–1.56 (m, 1H), 1.59 (s, 5H), 1.55 (d, *J* = 2.0 Hz, 1H), 1.51 (ddd, *J* = 12.6, 4.5, 2.7 Hz, 1H), 1.37 (s, 3H), 1.26 (d, *J* = 14.2 Hz, 1H), 1.10 (dddd, *J* = 26.5, 12.2, 8.5, 5.9 Hz, 2H), 0.97 (s, 3H), 0.91 (d, *J* = 6.8 Hz, 3H), 0.90 (s, 3H).

^13^C NMR (126 MHz, CDCl_3_) δ 170.9, 170.5, 148.3, 132.7, 130.5, 123.2, 77.4, 74.6, 71.5, 68.4, 51.5, 49.3, 48.8, 44.0, 39.6, 39.2, 37.2, 36.3, 35.7, 32.5, 30.4, 30.1, 29.1, 28.4, 25.9, 24.3, 22.9, 21.1, 20.9, 17.9, 17.9, 16.1.

Spectral data for **2a** matched those reported in the literature [49].

**4-methoxybenzyl(*Z*)-2-((3*R*,4*S*,5*S*,8*S*,9*S*,10*S*,11*R*,13*R*,14*S*,16*S*)-16-acetoxy-3,11-dihydroxy-4,8,10,14-tetramethylhexadecahydro-17H-cyclopenta[a]phenanthren-17-ylidene)-6-methylhept-5-enoate (2b)**

4-Methoxybenzyl alcohol (21 mg, 0.15 mmol, 1.5 equiv), EDCI (28 mg, 0.15 mmol, 1.5 equiv), and DMAP (12 mg, 0.1 mmol, 1 equiv) were added to a solution of fusidic acid (50 mg, 0.1 mmol, 1.0 equiv) in anhydrous DCM (1 mL). The reaction mixture was stirred at 25 °C under a N_2_ atmosphere. After the reaction was determined to be complete by TLC (24 h), the reaction mixture was diluted with ethyl acetate (10 mL), washed with H_2_O (5 mL), and saturated with aqueous brine (5 mL). The organic layer was then dried over anhydrous MgSO_4_, filtered, and concentrated in vacuo. The crude product was then purified by column chromatography (27.5% EA in hexane) on silica gel to afford the product **2b** (white solid, 31 mg, 49%). *R_f_* = 0.5 (50% EA in hexane):

^1^H NMR (500 MHz, CDCl_3_) δ 7.29 (dd, *J* = 8.5, 1.9 Hz, 2H), 6.87 (dd, *J* = 8.6, 1.9 Hz, 2H), 5.86 (d, *J* = 8.3 Hz, 1H), 5.13 (dd, *J* = 11.8, 1.7 Hz, 1H), 5.06–5.03 (m, 1H), 4.85 (dd, *J* = 11.9, 1.7 Hz, 1H), 4.32 (d, *J* = 2.7 Hz, 1H), 3.80 (d, *J* = 1.7 Hz, 3H), 3.74 (t, *J* = 2.6 Hz, 1H), 3.01 (d, *J* = 12.2 Hz, 1H), 2.43 (qt, *J* = 13.9, 6.8 Hz, 2H), 2.29 (dd, *J* = 13.2, 3.4 Hz, 1H), 2.12 (ddd, *J* = 33.3, 12.2, 6.0 Hz, 2H), 1.98 (dd, *J* = 15.1, 8.1 Hz, 1H), 1.93 (d, *J* = 1.8 Hz, 3H), 1.82 (tt, *J* = 12.8, 2.3 Hz, 2H), 1.76–1.70 (m, 2H), 1.63 (s, 3H), 1.58 (ddd, *J* = 11.9, 6.9, 2.1 Hz, 1H), 1.54 (t, *J* = 2.1 Hz, 1H), 1.51 (s, 3H), 1.35 (d, *J* = 1.8 Hz, 3H), 1.28 (d, *J* = 14.3 Hz, 1H), 1.25 (d, *J* = 2.0 Hz, 2H), 1.11 (tdd, *J* = 19.0, 9.1, 4.3 Hz, 2H), 0.96 (d, *J* = 1.8 Hz, 3H), 0.90 (s, 6H), 0.90 (dd, *J* = 18.6, 2.0 Hz, 2H).

^13^C NMR (126 MHz, CDCl_3_) δ 170.6, 170.2, 159.8, 148.2, 132.6, 130.6, 130.4, 128.0, 123.2, 114.1, 77.4, 74.6, 71.5, 68.4, 66.3, 55.4, 49.3, 48.8, 44.0, 39.6, 39.1, 37.2, 36.3, 36.3, 35.7, 32.5, 31.7, 30.4, 30.1, 29.2, 28.4, 25.8, 24.3, 22.9, 21.1, 20.9, 18.0, 17.8, 16.1, 14.3.

HRMS (ESI) *m*/*z* calculated for C_39_H_56_O_7_^+^ M + Na^+^: 659.3918; found: 659.3965.

[α]_D_^26^ = −1.5° (*c* = 0.68, CH_2_Cl_2_)

Melting point: 193.6 °C

**pyridin-2-ylmethyl(Z)-2-((** **3*R*,4*S*,5*S*,8*S*,9*S*,10*S*,11*R*,13*R*,14*S*,16*S*)-16-acetoxy-3,11-dihydroxy-4,8,10,14-tetramethylhexadecahydro-17H-cyclopenta[a]phenanthren-17-ylidene)-6-methylhept-5-enoate (2c)**

2-Pyridinemethanol (10.5 μL, 0.15 mmol, 1.5 equiv), EDCI (28 mg, 0.15 mmol, 1.5 equiv), and DMAP (12 mg, 0.1 mmol, 1 equiv) were added to a solution of fusidic acid (50 mg, 0.1 mmol, 1.0 equiv) in anhydrous DCM (1 mL). The reaction mixture was stirred at 25 °C under a N_2_ atmosphere. After the reaction was determined to be complete by TLC (24 h), the reaction mixture was diluted with ethyl acetate (10 mL), washed with H_2_O (5 mL), and saturated with aqueous brine (5 mL). The organic layer was then dried over anhydrous MgSO_4_, filtered, and concentrated in vacuo. The crude product was then purified by column chromatography (42.5% EA in hexane) on silica gel to afford the product **2c** (colorless solid, 36 mg, 60%). *R_f_* = 0.4 (60% EA in hexane):

^1^H NMR (500 MHz, CDCl_3_) δ 8.57 (d, *J* = 4.5 Hz, 1H), 7.70 (td, *J* = 7.7, 1.8 Hz, 1H), 7.37 (d, *J* = 7.8 Hz, 1H), 7.22 (dd, *J* = 7.6, 4.9 Hz, 1H), 5.88 (d, *J* = 8.4 Hz, 1H), 5.31 (d, *J* = 13.5 Hz, 1H), 5.08 (d, *J* = 13.5 Hz, 2H), 4.34 (q, *J* = 2.7 Hz, 1H), 3.74 (q, *J* = 2.8 Hz, 1H), 3.06 (dd, *J* = 12.8, 3.4 Hz, 1H), 2.49 (m, 2H), 2.31 (dt, *J* = 13.1, 3.3 Hz, 1H), 2.18 (dd, *J* = 14.5, 7.4 Hz, 2H), 2.07 (dddd, *J* = 30.4, 22.9, 12.5, 6.1 Hz, 2H), 1.94 (s, 3H), 1.85 (ddd, *J* = 15.4, 11.2, 2.8 Hz, 2H), 1.74 (tt, *J* = 7.7, 5.4 Hz, 2H), 1.63 (d, *J* = 1.2 Hz, 4H), 1.58 (dq, *J* = 10.5, 4.0 Hz, 1H), 1.55 (d, *J* = 2.0 Hz, 1H), 1.54 (d, *J* = 1.3 Hz, 3H), 1.50 (dq, *J* = 12.7, 2.7 Hz, 1H), 1.37 (s, 3H), 1.28 (d, *J* = 14.3 Hz, 1H), 1.26–1.24 (m, 1H), 1.09 (dddd, *J* = 26.7, 12.4, 8.6, 5.9 Hz, 2H), 0.97 (s, 3H), 0.91 (t, *J* = 3.4 Hz, 6H).

^13^C NMR (126 MHz, CDCl_3_) δ 170.6, 169.6, 155.7, 149.5, 148.9, 137.5, 132.7, 130.1, 123.2, 122.1, 77.4, 74.5, 71.5, 68.4, 66.6, 49.4, 48.8, 44.2, 39.6, 39.2, 37.2, 36.4, 36.2, 35.7, 32.5, 30.4, 30.1, 29.2, 28.6, 25.8, 24.3, 23.0, 21.1, 20.9, 18.0, 17.9, 16.1.

HRMS (ESI) *m*/*z* calculated for C_37_H_53_NO_6_^+^ M + H^+^: 608.3946; found: 608.3979; M + Na^+^: 630.3765; found: 630.3797.

[α]_D_^26^ = −3.0 (*c* = 1.64, CH_2_Cl_2_)

Melting point: 115.8 °C

**(Z)-2-((4** ***S*,5*S*,8*S*,9*S*,10*S*,11*R*,13*R*,14*S*,16*S*)-16-acetoxy-11-hydroxy-4,8,10,14-tetramethyl-3-oxohexadecahydro-17H-cyclopenta[a]phenanthren-17-ylidene)-6-methylhept-5-enoic acid (3a)**

**(Z)-2-((4** ***S*,5*S*,8*S*,9*S*,10*S*,13*R*,14*S*,16*S*)-16-acetoxy-4,8,10,14-tetramethyl-3,11-dioxohexadecahydro-17H-cyclopenta[a]phenanthren-17-ylidene)-6-methylhept-5-enoic acid (4a)**

Fusidic acid (100 mg, 0.2 mmol, 1 equiv) was dissolved in anhydrous dichloromethane (2.5 mL) while stirring under nitrogen at −10 °C. Dess–Martin periodinane (0.74 mg, 0.17 mmol, 0.9 equiv) was added and the mixture stirred for 3 h. The mixture was diluted with DCM (20 mL) and washed with saturated NaHCO_3_, brine (25 mL each), and dried (Na_2_SO_4_). The organic layer was filtered and reduced in vacuo to leave a clear film. The crude product was then purified by column chromatography (30% (**3a**), 27.5% (**4a**), EA in hexane) on silica gel to afford the product **3a** (colorless solid, 30 mg, 30%) and **4a** (light-yellow solid, 4 mg, 3.7%). *R_f_* = 0.5 (**3a**), 0.6 (**4a**) (50% EA in hexane):

**3a:**

^1^H NMR (500 MHz, CDCl_3_) δ 5.92 (d, *J* = 8.3 Hz, 1H), 5.12 (t, *J* = 7.1 Hz, 1H),4.42 (m, 1H), 3.07 (d, *J* = 12.2 Hz 1H), 2.55−2.44 (m, 4H,), 2.43−2.25 (m, 3H), 2.23−2.01 (m, 4H), 1.98 (s, 3H), 2.01−1.86 (m, 4H), 1.68 (s, 3H),1.67−1.64 (m, 1H), 1.62 (s, 3H), 1.35−1.33 (m, 1H), 1.33 (s, 3H), 1.27−1.13 (m, 2H), 1.18 (s, 3H), 1.04 (d, *J* = 6.6 Hz, 3H), 0.95 (s, 3H);

^13^C NMR (126 MHz, CDCl_3_) δ 213.9, 174.6, 170.7, 151.2, 132.8, 130.0, 123.0, 74.4, 68.2, 48.9, 48.5, 45.8, 45.5, 44.4, 39.5, 39.1, 38.5, 37.0, 36.25, 35.5, 33.7, 28.8, 28.5, 25.8, 24.9, 22.8, 22.0, 20.7, 18.2, 17.9.

**4a:**

^1^H NMR (500 MHz, CDCl_3_) δ 5.93 (d, *J* = 8.3 Hz, 1H), 5.08 (t, *J* = 7.2 Hz, 1H), 2.96–2.86 (m, 2H), 2.78–2.65 (m, 2H), 2.61 (s, 1H), 2.51(ddd, *J* = 13.7, 8.8, 4.7 Hz, 1H), 2.46–2.32 (m, 3H), 2.27–2.09 (m, 3H), 2.09–1.97 (m, 3H), 2.0 (s, 3H), 1.81 (t, *J* = 12.1 Hz,1H), 1.67 (s, 3H), 1.60 (s, 3H), 1.60–1.55 (m, 1H), 1.46 (d, J = 14.5 Hz, 1H), 1.26–1.18 (m, 1H), 1.20 (s, 3H), 1.18–1.1 (m, 1H), 1.15 (s, 3H), 1.06–1.02 (m, 6H).

^13^C NMR (126 MHz, CDCl_3_) δ 216.0, 209.6, 174.2, 170.4, 148.2, 133.3, 131.0, 122.6, 74.3, 58.4, 48.8, 47.1, 46.3, 44.9, 44.5, 40.9, 38.1, 36.8, 36.7, 33.4, 32.5, 28.9, 28.1, 25.8, 24.0, 22.6, 21.3, 20.6, 17.9, 17.1, 14.2.

Spectral data for **3a** and **4a** matched those reported in the literature [50].

**(Z)-2-((4*S*,5*S*,8*S*,9*S*,10*S*,13*R*,14*S*,16*S*)-16-acetoxy-4,8,10,14-tetramethyl-3,11-dioxohexadecahydro-17H-cyclopenta[a]phenanthren-17-ylidene)-6-methylhept-5-enoic acid (4a)**

Fusidic acid (100 mg, 0.2 mmol, 1 equiv) was dissolved in anhydrous dichloromethane (2.5 mL) while stirring under nitrogen at RT. Dess–Martin periodinane (191 mg, 0.45 mmol, 2.25 equiv) was added and the mixture stirred for 15 h. The mixture was diluted with DCM (20 mL) and washed with saturated NaHCO_3_, brine (25 mL each), and dried (Na_2_SO_4_). The organic layer was filtered and reduced in vacuo to leave a clear film. The crude product was then purified by column chromatography 27.5% EA in hexane on silica gel to afford the product **4a** (light-yellow solid, 80 mg, 80%). *R_f_* = 0.6 (50% EA in hexane):

^1^H NMR (500 MHz, CDCl_3_) δ 5.93 (d, *J* = 8.3 Hz, 1H), 5.08 (t, *J* = 7.2 Hz, 1H), 2.96–2.86 (m, 2H), 2.78–2.65 (m, 2H), 2.61 (s, 1H), 2.51 (ddd, *J*= 13.7, 8.8, 4.7 Hz, 1H), 2.46–2.32 (m, 3H), 2.27–2.09 (m, 3H), 2.09–1.97 (m, 3H), 2.0 (s, 3H), 1.81 (t, *J*= 12.1 Hz,1H), 1.67 (s, 3H), 1.60 (s, 3H), 1.60–1.55 (m, 1H), 1.46 (d, *J* = 14.5 Hz, 1H), 1.26–1.18 (m, 1H), 1.20 (s, 3H), 1.18–1.1 (m, 1H), 1.15 (s, 3H), 1.06–1.02 (m, 6H).

^13^C NMR (126 MHz, CDCl_3_) δ 216.0, 209.6, 174.2, 170.4, 148.2, 133.3, 131.0, 122.6, 74.3, 58.4, 48.8, 47.1, 46.3, 44.9, 44.5, 40.9, 38.1, 36.8, 36.7, 33.4, 32.5, 28.9, 28.1, 25.8, 24.0, 22.6, 21.3, 20.6, 17.9, 17.1, 14.2.

Spectral data for **4a** matched those reported in the literature [50].

**(Z)-2-((4** ***S*,5*S*,8*S*,9*S*,10*S*,11*R*,13*R*,14*S*,16*S*,*E*)-16-acetoxy-11-hydroxy-3-(hydroxyimino)-4,8,10,14-tetramethylhexadecahydro-17H-cyclopenta[a]phenanthren-17-ylidene)-6-methylhept-5-enoic acid (3b)**

**3a** (103.0 mg, 0.2 mmol, 1 equiv) was dissolved in isopropanol (1 mL) while stirring under nitrogen, then 50% aqueous hydroxyamine (49 μL 0.8 mmol, 4 equiv) and acetic acid (46 μL, 0.8 mmol, 4 equiv) were added and the mixture warmed to 50 °C and stirred for 15 h. The mixture was reduced in vacuo to leave a clear film. The crude product was then purified by column chromatography (30% EA in DCM) on silica gel to afford the product **3b** (colorless solid, 62 mg, 60%). *R_f_* = 0.3 (30% EA in DCM):

^1^H NMR (500 MHz, CDCl_3_) δ 5.90 (dd, *J* = 11.5, 8.4 Hz, 1H), 5.10 (t, *J* = 7.2 Hz, 1H), 4.37 (s, 1H), 3.26–3.19 (m, 1H), 3.03 (d, *J* = 12.2 Hz, 1H), 2.68 (q, *J* = 9.5 Hz, 1H), 2.51–2.43 (m, 2H), 2.33–2.26 (m, 2H), 2.21–2.13 (m, 2H), 2.12–1.95 (m, 2H), 1.94 (d, *J* = 2.1 Hz, 3H), 1.90 (t, *J* = 12.3 Hz, 1H), 1.86–1.70 (m, 1H), 1.67 (s, 3H), 1.60 (s, 3H), 1.55 (d, *J* = 33.4 Hz, 1H), 1.31 (d, *J* = 23.0 Hz, 3H), 1.39–1.24 (m, 2H), 1.08 (dd, *J* = 36.3, 6.6 Hz, 3H), 1.07 (s, 2H), 0.93 (d, *J* = 4.0 Hz, 3H), 0.83 (s, 1H).

^13^C NMR (126 MHz, CDCl_3_) δ 174.9, 174.8, 170.7, 166.2, 163.6, 149.4, 149.0, 132.6, 130.6, 130.4, 123.1, 74.3, 68.1, 67.9, 48.9, 48.7, 48.6, 46.7, 45.2, 44.1, 43.9, 43.6, 39.4, 39.3, 38.9, 37.4, 36.8, 36.6, 36.2, 36.0, 35.4, 34.3, 33.5, 33.1, 32.2, 28.8, 28.3, 25.7, 24.3, 23.9, 22.7, 21.6, 21.2, 20.6, 20.5, 18.3, 17.8, 17.8, 14.2, 14.0.

Spectral data for **3b** matched those reported in the literature [40].

**(Z)-2-((3** ***E*,4*S*,5*S*,8*S*,9*S*,10*S*,11*E*,13*R*,14*S*,16*S*)-16-acetoxy-3,11-bis(hydroxyimino)-4,8,10,14-tetramethylhexadecahydro-17H-cyclopenta[a]phenanthren-17-ylidene)-6-methylhept-5-enoic acid (4b)**

**4a** (103.0 mg, 0.2 mmol, 1 equiv) was dissolved in isopropanol (1 mL) while stirring under nitrogen. Then, 50% aqueous hydroxyamine (61 μL. 1 mmol, 5 equiv) and acetic acid (58 μL, 1 mmol, 5 equiv) were added and the mixture warmed to 50 °C and stirred for 15 h. The crude was reduced in vacuo to leave a clear film. The crude product was then purified by column chromatography (30% EA in DCM) on silica gel to afford the product **4b** (colorless solid, 74 mg, 70%). *R_f_* = 0.25 (30% EA in DCM):

^1^H NMR (500 MHz, CDCl_3_) δ 5.65 (d, *J* = 8.5 Hz, 1H), 4.94 (t, *J* = 7.4 Hz, 1H), 3.79 (dq, *J* = 15.5, 5.5 Hz, 1H), 3.22–3.12 (m, 4H), 2.50 (tt, *J* = 12.8, 5.4 Hz, 1H), 2.34 (dd, *J* = 13.9, 5.9 Hz, 2H), 2.26–2.20 (m, 1H), 2.14 (td, *J* = 10.0, 3.3 Hz, 1H), 2.06–1.93 (m, 1H), 1.88 (dd, *J* = 13.4, 5.4 Hz, 1H), 1.84–1.81 (m, 1H), 1.80 (d, *J* = 3.5 Hz, 3H), 1.77–1.70 (m, 1H), 1.52 (t, *J* = 11.6 Hz, 1H), 1.44 (s, 3H), 1.39 (s, 3H), 1.32 (dd, *J* = 12.0, 5.0 Hz, 1H), 1.13 (dd, *J* = 14.4, 3.4 Hz, 1H), 1.05 (t, *J* = 3.3 Hz, 1H), 0.98–0.92 (m, 1H), 0.89 (s, 3H), 0.88 (s, 2H), 0.88 (s, 1H), 0.87 (s, 2H), 0.82 (d, *J* = 3.2 Hz, 3H), 0.77 (d, *J* = 3.2 Hz, 3H).

^13^C NMR (126 MHz, CDCl_3_) δ 172.4, 171.5, 167.0, 156.6, 146.1, 132.6, 131.7, 122.9, 74.3, 50.2, 45.0, 44.4, 39.4, 38.1, 36.5, 33.7, 33.2, 32.4, 28.8, 27.9, 25.6, 24.8, 24.3, 23.7, 22.7, 20.4, 20.1, 17.5, 16.6, 14.2.

Spectral data for **4b** matched those reported in the literature [40].

**(Z)-2-((** **3*R*,4*S*,5*S*,8*S*,9*S*,10*S*,11*R*,13*R*,14*S*,16*S*)-16-acetoxy-3-(benzyloxy)-11-hydroxy-4,8,10,14-tetramethylhexadecahydro-17H-cyclopenta[a]phenanthren-17-ylidene)-6-methylhept-5-enoic acid (5a)**

Fusidic acid (100 mg, 0.2 mmol, 1.0 equiv) was added to a premixed solution of benzoic acid (38 mg, 0.4 mmol, 2.0 equiv), EDCI (75 mg, 0.4 mmol, 2.0 equiv), and DMAP (30 mg, 0.3 mmol, 1.25 equiv) in anhydrous DCM (0.6 mL). The reaction mixture was stirred at 25 °C under a N_2_ atmosphere. After the reaction was determined to be complete by TLC (24 h), the reaction mixture was diluted with ethyl acetate (10 mL), washed with H_2_O (5 mL), and saturated with aqueous brine (5 mL). The organic layer was then dried over anhydrous MgSO_4_, filtered, and concentrated in vacuo. The crude product was then purified by column chromatography (25% EA in hexane) on silica gel to afford the product **5a** (colorless solid, 29 mg, 24%). *R_f_* = 0.5 (50% EA in hexane):

^1^H NMR (500 MHz, CDCl_3_) δ 8.05 (dd, *J* = 8.3, 1.4 Hz, 2H), 7.56 (tt, *J* = 6.9, 1.4 Hz, 1H), 7.45 (t, *J* = 7.7 Hz, 2H), 5.91 (d, *J* = 8.3 Hz, 1H), 5.20 (d, *J* = 2.7 Hz, 1H), 5.10 (ddd, *J* = 8.7, 4.5, 1.8 Hz, 1H), 4.36 (q, *J* = 2.8 Hz, 1H), 3.08 (dd, *J* = 12.7, 3.2 Hz, 1H), 2.46 (dh, *J* = 19.2, 6.5 Hz, 2H), 2.36–2.29 (m, 2H), 2.20 (dt, *J* = 23.9, 7.2 Hz, 3H), 2.12–1.99 (m, 1H), 1.97 (s, 3H), 1.92 (dt, *J* = 13.0, 3.1 Hz, 2H), 1.89–1.78 (m, 2H), 1.68 (d, *J* = 8.9 Hz, 2H), 1.67 (s, 3H), 1.59 (d, *J* = 1.4 Hz, 3H), 1.46 (s, 3H), 1.35 (d, *J* = 14.4 Hz, 1H), 1.25 (s, 2H), 1.24–1.07 (m, 2H), 1.04 (s, 3H), 0.94 (s, 3H), 0.90 (d, *J* = 6.8 Hz, 3H).

^13^C NMR (126 MHz, CDCl_3_) δ 174.6, 170.9, 166.3, 150.86, 132.9, 132.7, 131.0, 129.9, 129.6, 128.6, 123.2, 75.1, 74.6, 68.2, 49.1, 48.9, 44.4, 39.6, 39.1, 38.2, 37.1, 35.9, 35.2, 33.0, 31.2, 29.8, 28.8, 28.5, 27.5, 25.8, 25.8, 24.6, 22.7, 20.7, 20.7, 18.2, 17.9, 15.9.

HRMS (ESI) *m*/*z* calculated for C_38_H_52_O_7_^+^ M + Na^+^: 643.3605; found: 643.3657.

IR (neat, cm^−1^) 2928, 1713, 1451, 1377, 1271, 1115, 1027, 737, 713.

[α]_D_^20^ = −20.5 (*c* = 0.41, CH_2_Cl_2_)

Melting point: 114.2 °C

**(Z)-2-((** **3*R*,4*S*,8*S*,9*S*,10*S*,11*R*,13*S*,14*S*,16*S*)-16-acetoxy-11-hydroxy-4,8,10,14-tetramethyl-3-(picolinoyloxy) hexadecahydro-17H-cyclopenta[a]phenanthren-17-ylidene)-6-methylhept-5-enoic acid (5b)**

Fusidic acid (100 mg, 0.2 mmol, 1.0 equiv) was added to a premixed solution of picolinic acid (48 mg, 0.4 mmol, 2.0 equiv), EDCI (74 mg, 0.4 mmol, 2.0 equiv), and DMAP (30 mg, 0.2 mmol, 1.25 equiv) in anhydrous DMF (0.6 mL). The reaction mixture was stirred at 25 °C under a N_2_ atmosphere. After the reaction was determined to be complete by TLC (24 h), the reaction mixture was diluted with ethyl acetate (10 mL), washed with H_2_O (5 mL), and saturated with aqueous brine (5 mL). The organic layer was then dried over anhydrous MgSO_4_, filtered, and concentrated in vacuo. The crude product was then purified by column chromatography (2% MeOH in DCM) on silica gel to afford the product **5b** (colorless solid, 50 mg, 41%). *R_f_* = 0.3 (60% EA in hexane):

^1^H NMR (500 MHz, CDCl_3_) δ 8.83 (d, *J* = 5.5 Hz, 1H), 8.09 (d, *J* = 7.8 Hz, 1H), 7.89 (td, *J* = 7.7, 1.8 Hz, 1H), 7.51 (dd, *J* = 7.7, 4.7 Hz, 1H), 5.93 (d, *J* = 8.4 Hz, 1H), 5.32 (q, *J* = 2.9 Hz, 1H), 5.12 (t, *J* = 7.2 Hz, 1H), 4.39 (q, *J* = 2.6 Hz, 1H), 3.10 (dd, *J* = 12.4, 3.2 Hz, 1H), 2.48 (q, *J* = 8.0 Hz, 2H), 2.35 (td, *J* = 11.5, 4.3 Hz, 2H), 2.25 (ddt, *J* = 22.4, 14.7, 6.2 Hz, 2H), 2.20–2.15 (m, 1H), 2.12–2.04 (m, 1H), 2.03–1.99 (m, 1H), 1.97 (s, 3H), 1.95–1.87 (m, 1H), 1.84 (ddd, *J* = 11.8, 7.2, 3.5 Hz, 2H), 1.68 (s, 3H), 1.62 (d, *J* = 9.8 Hz, 6H), 1.47 (s, 3H), 1.36 (d, *J* = 14.2 Hz, 1H), 1.27 (s, 1H), 1.25–1.11 (m, 2H), 1.06 (s, 3H), 0.96 (s, 3H), 0.93 (d, *J* = 6.7 Hz, 3H).

^13^C NMR (126 MHz, CDCl_3_) δ 174.5, 170.7, 163.7, 150.7, 149.6, 147.7, 138.4, 132.7, 129.9, 127.2, 125.2, 123.2, 77.4, 77.2, 76.9, 76.6, 74.5, 68.2, 49.3, 48.9, 44.4, 39.6, 39.1, 37.9, 37.0, 35.9, 35.4, 32.5, 31.1, 28.9, 28.6, 25.8, 24.4, 23.0, 20.9, 20.7, 18.1, 17.9, 16.0, 15.9.

HRMS (ESI) *m*/*z* calculated for C_37_H_51_NO_7_^+^ M + H^+^: 622.3738; found: 622.3778; M + Na^+^: 644.3558; found: 644.3589.

IR (neat, cm^−1^) 2932, 1735, 1438, 1378, 1246, 1141, 1031, 748.

[α]_D_^20^ = −18.7 (*c* = 0.3, CH_2_Cl_2_)

Melting point: 130.2 °C

**(Z)-2-((** **3*R*,4*S*,8*S*,9*S*,10*S*,11*R*,13*S*,14*S*,16*S*)-16-acetoxy-11-hydroxy-4,8,10,14-tetramethyl-3-(nicotinoyloxy)hexadecahydro-17H-cyclopenta[a]phenanthren-17-ylidene)-6-methylhept-5-enoic acid (5c)**

Fusidic acid (100 mg, 0.2 mmol, 1.0 equiv) was added to a premixed solution of nicotinic acid (48 mg, 0.4 mmol, 2.0 equiv), EDCI (74 mg, 0.4 mmol, 2.0 equiv), and DMAP (30 mg, 0.2 mmol, 1.25 equiv) in anhydrous DCM (0.6 mL). The reaction mixture was stirred at 25 °C under a N_2_ atmosphere. After the reaction was determined to be complete by TLC (24 h), the reaction mixture was diluted with ethyl acetate (10 mL), washed with H_2_O (5 mL), and saturated with aqueous brine (5 mL). The organic layer was then dried over anhydrous MgSO_4_, filtered, and concentrated in vacuo. The crude product was then purified by column chromatography (25% EA in hexane) on silica gel to afford the product **5c** (colorless solid, 68 mg, 56%). *R_f_* = 0.3 (60% EA in hexane):

^1^H NMR (500 MHz, CDCl_3_) δ 9.32 (s, 1H), 8.89 (s, 1H), 8.49 (d, *J* = 7.9 Hz, 1H), 7.58 (s, 1H), 5.98 (d, *J* = 8.3 Hz, 1H), 5.28 (q, *J* = 2.8 Hz, 1H), 5.11 (t, *J* = 7.3 Hz, 1H), 4.35 (d, *J* = 2.8 Hz, 1H), 3.07 (dd, *J* = 12.5, 3.2 Hz, 1H), 2.47 (t, *J* = 8.0 Hz, 2H), 2.40 (td, *J* = 11.7, 4.8 Hz, 1H), 2.36–2.27 (m, 2H), 2.20 (dt, *J* = 15.4, 7.8 Hz, 2H), 2.14–2.00 (m, 2H), 1.92 (tt, *J* = 13.2, 6.8 Hz, 3H), 1.86 (s, 3H), 1.83–1.76 (m, 2H), 1.66 (s, 3H), 1.60 (s, 3H), 1.52 (s, 3H), 1.34 (d, *J* = 14.2 Hz, 1H), 1.25 (q, *J* = 6.1 Hz, 3H), 1.13 (tt, *J* = 13.5, 7.3 Hz, 1H), 1.04 (s, 3H), 0.95 (s, 3H), 0.92 (d, *J* = 6.7 Hz, 3H).

^13^C NMR (126 MHz, CDCl_3_) δ 173.6, 170.5, 164.0, 151.0, 148.9, 148.7, 139.4, 132.4, 130.7, 124.4, 123.3, 123.0, 77.3, 77.2, 77.0, 76.8, 76.5, 74.4, 68.3, 49.2, 48.9, 43.9, 39.5, 39.1, 37.8, 36.9, 35.9, 35.2, 32.3, 31.1, 29.7, 28.9, 28.5, 27.5, 25.8, 24.4, 22.8, 20.7, 20.7, 17.9, 15.8.

HRMS (ESI) *m*/*z* calculated for C_37_H_51_NO_7_^+^ M + H^+^: 622.3738; found: 622.3784; M + Na^+^: 644.3558; found: 644.3601.

IR (neat, cm^−1^) 2932, 1720, 1377, 1284, 1125, 1086, 1030, 740, 702.

[α]_D_^20^ = −16.8 (*c* = 0.7, CH_2_Cl_2_)

Melting point: 136.2 °C

**(Z)-2-((** **3*R*,4*S*,5*S*,8*S*,9*S*,10*S*,11*R*,13*R*,14*S*,16*S*)-16-acetoxy-11-hydroxy-3-(isonicotinoyloxy)-4,8,10,14-tetramethylhexadecahydro-17H-cyclopenta[a]phenanthren-17-ylidene)-6-methylhept-5-enoic acid (5d)**

Fusidic acid (100 mg, 0.2 mmol, 1.0 equiv) was added to a premixed solution of isonicotinic acid (48 mg, 0.4 mmol, 2.0 equiv), EDCI (74 mg, 0.4 mmol, 2.0 equiv), and DMAP (30 mg, 0.2 mmol, 1.25 equiv) in anhydrous DMF (0.6 mL). The reaction mixture was stirred at 25 °C under a N_2_ atmosphere. After the reaction was determined to be complete by TLC (24 h), the reaction mixture was diluted with EA (10 mL), washed with H_2_O (5 mL), and saturated with aqueous brine (5 mL). The organic layer was then dried over anhydrous MgSO_4_, filtered, and concentrated in vacuo. The crude product was then purified by column chromatography (1.75% MeOH in DCM) on silica gel to afford the product **5d** (colorless solid, 55 mg, 45%). *R_f_* = 0.3 (60% EA in hexane):

^1^H NMR (500 MHz, CDCl_3_) δ 8.89 (d, *J* = 5.4 Hz, 2H), 8.09 (d, *J* = 5.2 Hz, 2H), 5.80 (d, *J* = 8.3 Hz, 1H), 5.26 (q, *J* = 2.7 Hz, 1H), 5.14–5.07 (m, 1H), 4.39–4.34 (m, 1H), 3.10 (t, *J* = 8.6 Hz, 1H), 2.50–2.18 (m, 4H), 2.11 (dq, *J* = 14.4, 7.6 Hz, 1H), 2.04 (s, 3H), 2.00–1.89 (m, 3H), 1.82 (dtt, *J* = 19.9, 7.9, 3.9 Hz, 2H), 1.68 (s, 3H), 1.63–1.61 (m, 3H), 1.60 (d, *J* = 2.0 Hz, 2H), 1.45 (s, 3H), 1.41 (t, *J* = 7.3 Hz, 1H), 1.35 (d, *J* = 14.3 Hz, 1H), 1.25 (s, 1H), 1.24–1.11 (m, 2H), 1.05 (s, 3H), 0.95 (s, 3H), 0.90 (d, *J* = 6.7 Hz, 3H).

^13^C NMR (176 MHz, CDCl_3_) δ 171.0, 164.7, 163.5, 153.0, 132.7, 129.2, 123.0, 74.2, 68.1, 49.0, 48.8, 45.8, 44.6, 44.0, 39.6, 39.6, 39.1, 38.0, 36.9, 36.0, 35.2, 32.4, 30.9, 29.7, 29.1, 29.0, 27.3, 25.8, 24.4, 22.8, 21.2, 20.7, 18.1, 17.9, 15.9, 8.6.

HRMS (ESI) *m*/*z* calculated for C_37_H_51_NO_7_^+^ M + Na^+^: 644.3558; found: 644.3603.

IR (neat, cm^−1^) 2961, 2880, 1726, 1377, 1282, 1124, 1031, 483, 418,

[α]_D_^20^ = −14.4 (*c* = 0.46, CH_2_Cl_2_)

Melting point: 123.5 °C

**(Z)-2-((** **3*R*,4*S*,5*S*,8*S*,9*S*,10*S*,11*R*,13*R*,14*S*,16*S*)-16-acetoxy-11-hydroxy-4,8,10,14-tetramethyl-3-((pyrazine-2-carbonyl)oxy)hexadecahydro-17H-cyclopenta[a]phenanthren-17-ylidene)-6-methylhept-5-enoic acid (5e)**

Fusidic acid (100 mg, 0.2 mmol, 1.0 equiv) was added to a premixed solution of pyrazinoic acid (48 mg, 0.4 mmol, 2.0 equiv), EDCI (74 mg, 0.4 mmol, 2.0 equiv), and DMAP (30 mg, 0.2 mmol, 1.25 equiv) in anhydrous DMF (0.6 mL). The reaction mixture was stirred at 25 °C under a N_2_ atmosphere. After the reaction was determined to be complete by TLC (24 h), the reaction mixture was diluted with ethyl acetate (10 mL), washed with H_2_O (5 mL), and saturated with aqueous brine (5 mL). The organic layer was then dried over anhydrous MgSO_4_, filtered, and concentrated in vacuo. The crude product was then purified by column chromatography (3% MeOH in DCM) on silica gel to afford the product **5e** (colorless solid, 55 mg, 45%). *R_f_* = 0.5 (60% EA in hexane):

^1^H NMR (500 MHz, CDCl_3_) δ 9.29 (d, *J* = 1.4 Hz, 1H), 8.77 (t, *J* = 1.9 Hz, 1H), 8.76 (d, *J* = 2.4 Hz, 1H), 5.92 (d, *J* = 8.4 Hz, 1H), 5.33 (q, *J* = 2.8 Hz, 1H), 5.10 (t, 1H), 4.36 (q, *J* = 2.7 Hz, 1H), 3.07 (dd, *J* = 12.8, 3.2 Hz, 1H), 2.52–2.41 (m, 2H), 2.38–2.26 (m, 2H), 2.26–2.13 (m, 2H), 2.11–2.01 (m, 1H), 1.99 (dd, *J* = 5.6, 3.0 Hz, 1H), 1.96 (s, 3H), 1.91 (dd, *J* = 12.8, 2.7 Hz, 1H), 1.89–1.79 (m, 2H), 1.72–1.68 (m, 1H), 1.67–1.66 (m, 5H), 1.65–1.60 (m, 2H), 1.59 (d, *J* = 1.3 Hz, 3H), 1.46 (s, 3H), 1.35 (d, *J* = 14.3 Hz, 1H), 1.26–1.09 (m, 2H), 1.05 (s, 3H), 0.94 (s, 3H), 0.93 (d, *J* = 6.7 Hz, 3H).

^13^C NMR (126 MHz, CDCl_3_) δ 173.8, 170.6, 163.4, 151.0, 147.3, 145.9, 145.0, 144.4, 132.8, 129.7, 123.1, 77.4, 77.1, 74.5, 68.4, 49.2, 49.0, 44.4, 39.7, 39.1, 38.1, 37.1, 36.0, 35.3, 32.7, 31.2, 28.9, 28.5, 27.5, 25.9, 24.5, 22.9, 20.8, 18.2, 17.9, 16.0.

HRMS (ESI) *m*/*z* calculated for C_36_H_50_N_2_O_7_^+^ M + Na^+^: 645.3510; found: 645.3554.

IR (neat, cm^−1^) 2929, 2871, 2113, 1732, 1454, 1377, 1342, 1300, 1251, 1237, 1159, 1143, 1052, 1018, 974, 951, 880, 775, 736, 703, 653, 609, 505, 488, 462, 428.

[α]_D_^20^ = −20.0 (*c* = 0.27, CH_2_Cl_2_)

Melting point: 118.8 °C

**(Z)-2-((** **3*R*,4*S*,5*S*,8*S*,9*S*,10*S*,11*R*,13*R*,14*S*,16*S*)-16-acetoxy-11-hydroxy-4,8,10,14-tetramethyl-3-((oxazole-4-carbonyl)oxy)hexadecahydro-17H-cyclopenta[a]phenanthren-17-ylidene)-6-methylhept-5-enoic acid (5e)**

Oxazole-4-carboxylic acid (100 mg, 0.9 mmol, 1.0 equiv) was dissolved in 1 mL of anhydrous tetrahydrofuran (THF). Oxalyl chloride (152 μL, 1.8 mmol, 2.0 equiv) was added dropwise under an inert nitrogen atmosphere, followed by a catalytic amount of DMF (0.1 equiv). The reaction mixture was stirred at room temperature for 4 h. Upon completion, the reaction was monitored by TLC to ensure the formation of the acyl chloride intermediate. The solvent and excess oxalyl chloride were removed under reduced pressure, yielding a yellowish residue. Fusidic acid (153 mg, 0.3 mmol, 0.33 equiv) was dissolved in 1 mL of anhydrous DCM, followed by the addition of pyridine (36 μL, 0.5 mmol, 0.5 equiv) and DMAP (55 mg, 0.5 mmol, 0.5 equiv). The resulting solution was then added dropwise to the previously prepared acyl chloride intermediate under an inert nitrogen atmosphere at 0 °C. The reaction mixture was allowed to warm to room temperature and stirred overnight. After the reaction was determined to be complete by TLC (24 h), the reaction mixture was diluted with DCM (10 mL), washed with saturated NaHCO_3_ solution once (5 mL), and water twice (5 mL), and then saturated with aqueous brine (5 mL). The organic layer was then dried over anhydrous MgSO_4_, filtered, and concentrated in vacuo. The crude product was then purified by column chromatography (1.5% MeOH in DCM, 2nd column: 30% EA/hexane) on silica gel to afford the product **5e** (colorless solid, 42 mg, 35%). *R_f_* = 0.5 (60% EA in hexane):

^1^H NMR (500 MHz, CDCl_3_) δ 8.20 (d, *J* = 1.1 Hz, 1H), 7.95 (d, *J* = 1.0 Hz, 1H), 5.91 (d, *J* = 8.4 Hz, 1H), 5.21 (t, *J* = 2.7 Hz, 1H), 5.10 (t, 1H), 4.36 (d, *J* = 2.9 Hz, 1H), 3.07 (d, *J* = 12.3 Hz, 1H), 2.49 (d, *J* = 7.0 Hz, 1H), 2.46 (d, *J* = 6.6 Hz, 1H), 2.31 (dt, *J* = 13.2, 3.3 Hz, 1H), 2.28–2.20 (m, 2H), 2.19 (d, *J* = 6.5 Hz, 1H), 2.17–2.13 (m, 1H), 2.11–2.03 (m, 1H), 1.99 (d, *J* = 1.6 Hz, 1H), 1.98 (s, 3H), 1.91 (dd, *J* = 16.1, 3.5 Hz, 2H), 1.85–1.75 (m, 2H), 1.67 (d, *J* = 1.4 Hz, 3H), 1.60 (d, *J* = 1.4 Hz, 4H), 1.59–1.57 (m, 1H), 1.43 (s, 3H), 1.35 (d, *J* = 14.2 Hz, 1H), 1.26 (d, *J* = 2.4 Hz, 1H), 1.21–1.16 (m, 1H), 1.11 (dq, *J* = 13.9, 6.1 Hz, 1H), 1.02 (s, 3H), 0.94 (s, 3H), 0.89 (d, *J* = 6.7 Hz, 3H).

^13^C NMR (126 MHz, CDCl_3_) δ 173.0, 170.7, 160.6, 151.7, 151.2, 143.6, 133.7, 132.9, 129.5, 123.1, 75.6, 74.5, 68.4, 49.2, 49.0, 44.5, 39.7, 39.1, 38.0, 37.1, 36.0, 35.2, 32.8, 31.2, 28.9, 28.5, 27.5, 25.9, 24.6, 22.9, 20.8, 20.7, 18.2, 17.9, 15.9.

HRMS (ESI) *m*/*z* calculated for C_35_H_49_NO_8_^+^ M + Na^+^: 634.3350; found: 634.3394.

IR (neat, cm^−1^) 3462, 3137, 2929, 2873, 1722, 1578, 1517, 1455, 1378, 1253, 1165, 1102, 1056, 1031, 975, 911, 878, 768, 736, 649, 609

[α]_D_^25^ = −16.7° (*c* = 0.43, CH_2_Cl_2_)

Melting point: 195.1 °C

### 3.3. Antibacterial Activity Analysis

Bacterial strains tested were *Staphylococcus aureus* ATCC 25923 from the American Type Culture Collection (Manassas, VA, USA); *E. coli* K-12 BW25113 (CGSC #7636); *E. coli* K-12 JW5503-KanS *ΔtolC* (CGSC #14206); and *E. coli* K-12 RFM795 *lptD*4213 (CGSC #14179) from the Coli Genetics Stock Center (Yale University, New Haven, CT, USA). The *ΔtolC* strain has a deletion of the entire *tolC* gene except for nucleotides encoding the last 6 amino acids of this protein and can be considered a null mutant [46]. The *lptD*4213 mutation has also been called imp-1423 [24]. It consists of a deletion of amino acids 330–352 of the LptD protein. This well-characterized deletion mutant compromises the ability of bacteria to transport LPS into the outer membrane, decreasing the outer membrane permeability barrier.

Minimal inhibitory concentrations (MIC) were determined using Clinical Laboratory Standards Institute (CLSI) reference method equivalent technology as previously described [48]. The fusidic acid stock concentration was 48 mM in DMSO, while the fusidic analogue concentration was 50 mM in DMSO [48]. Compounds were dispensed with a Tecan D300 digital dispenser to create a doubling dilution series for each compound from 128 to 0.03125 μM. Assays were performed in 384-well format, and complete growth inhibition, i.e., the MIC, was scored based on A_600_ as previously described [48]. Values shown represent modal MICs from three technical replicates.

An *E. coli* S30 extract system for circular DNA (Promega, Madison, WI, USA) was used for coupled in vitro transcription–translation assays set up in 384-well black microplates (PHENIX Research, Swedesboro, NJ, USA). Nanoluciferase (NLuc, 100 ng) expression plasmid and compounds in concentrations ranging from 400 μM to 0.1 μM. in twofold dilutions were added to each reaction, followed by incubation at 37 °C for 60 min. After incubation, PBS containing a 1000-fold dilution of furimazine substrate (Nano-Glo Luciferase Assay System, Promega, Madison, WI, USA) was added to each well, and luminescence was measured using an Infinite M1000 Pro microplate reader (Tecan, Morrisville, NC, USA). Data were collected from three independent experiments. IC_50_ values were calculated from dose–response curves using a three-parameter nonlinear regression model (Prism 10, GraphPad, San Diego, CA, USA).

## 4. Conclusions

Three classes of fusidic acid analogues were prepared and screened for antibiotic activity. The analogues **2a**–**c**, which probed the effect of carboxylic acid ester functionalization, were inactive against *S. aureus* and *E. coli*. The analogues **3a/b** and **4a/b**, which probed the effect of alcohol oxidation and oxime formation in the A- and C-rings, showed a moderate tolerance for A-ring functionalization, with significant additional reduction in activity with either C-ring functionalization and/or oxime formation. The A-ring ester analogues **5a**–**f** showed a decrease in antibacterial activity against *S. aureus* and no activity against *E. coli*. Interestingly the antibacterial activity of the most active ester analogue, **5e**, was significantly greater than would be expected based on ribosome inhibitory activity in in vitro translation assays. This suggested that the pyrazine-2-carboxylate ester in the A-ring might spontaneously hydrolyze, conferring on **5e** properties of a fusidic acid prodrug. Investigation into the potential of A-ring esters to serve as prodrugs with enhanced pharmacological properties is ongoing and will be reported in due course.

## Figures and Tables

**Table 1:** Antibacterial activity (minimal inhibitory concentration in µM) of ester analogues against *Staphylococcus aureus* and *Escherichia coli* ^1^.

Compound	*S. aureus*	*E. coli* wt	*E. coli ΔtolC*	*E. coli lpt* *D* *4213*
FA (**1**)	0.125	>128	2	8
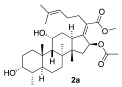	32	>128	>128	>128
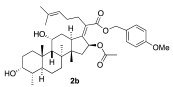	>128	>128	>128	>128
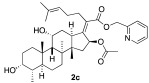	>128	>128	>128	>128

^1^ Minimal inhibitory concentration activity was determined through testing doubling dilutions of the indicated compounds. Values shown are the modal values of three replicate measurements.

**Table 2:** Effect of A-ring and C-ring modifications on prokaryotic in vitro translation (IC_50_) and antibacterial activity (MIC in µM).

Compound	In Vitro Translation IC_50_ (μM)	*S. aureus*	*E. coli* wt	*E. coli ΔtolC*	*E. coli lptD*4213
FA (**1**)	1.8	0.125	>128	2	8
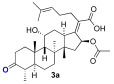	61.8	2 *	>128	16	>128
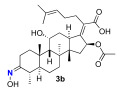	125.9	16	>128	4	128
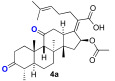	366.3	8	>128	>128	>128
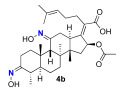	89.9	>128	>128	64	>128

Minimal inhibitory concentration and IC_50_ values for in vitro translation assays were determined through testing doubling dilutions of the indicated compounds. MIC values shown are the modal values for three technical replicates, except for *, where three different values were obtained for the 3 replicates and the median of the three values is shown. IC_50_ values are the mean of three independent determinations.

**Table 3:** Effect of aromatic carboxylic acid modification of A-ring on prokaryotic in vitro translation (IC_50_) and antibacterial activity (MIC in µM).

Compound	In Vitro Translation IC_50_ (μM)	*S. aureus*	*E. coli* wt	*E. coli ΔtolC*	*E. coli lptD*4213
FA (**1**)	1.8	0.125	>128	2	8
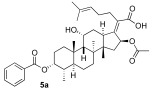	19.7	4	>64	64	>64
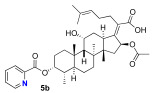	31.5	32	>64	64	>64
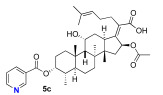	35.6	16	>128	>128	>128
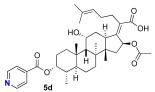	300.5	64 *	>64	>64	>64
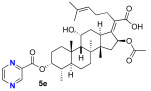	38.6	0.5	>128	64	>128
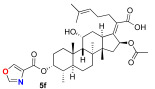	19.9	16	>128	>128	>128

Minimal inhibitory concentration and IC_50_ in in vitro translation assays were determined through testing doubling dilutions of the indicated compounds. MIC values shown are the modal values for three technical replicates. * Where three different values were obtained for the 3 replicates, the median of the three values is shown. IC_50_ values are the mean of three independent determinations.

## Data Availability

Full experimental procedures and spectral data are provide for all the new compounds reported (see Experimental Section and Supplementary Materials). The organisms used in this study are all commercially available.

## Supplementary Materials

Copies of ^1^H- and ^13^C{1H}-NMR spectra for all new compounds are available online at https://www.mdpi.com/article/10.3390/molecules30030465/s1.
