# Supplementary material for: Exploration of the Fusidic Acid Structure Activity Space for Antibiotic Activity
Source: Molecules. 2025 Jan 21;30(3):465. doi: 10.3390/molecules30030465 (PMC11820832; doi:10.3390/molecules30030465)
Supplement: Supplementary file 1 [file molecules-30-00465-s001.zip › molecules-3419491-supplementary.pdf]

Supporting Information

Exploration of the Fusidic Acid Structure Activity Space for Antibiotic activity

Yoon-Suk Kang†, Simone C. Silva†, Kenneth Smith†, Krissty Sumida†, Yuhan Wang†, Lucius Chiaraviglio*, Rama-chandra Reddy Donthiri*, Alhanouf Z. Aljahdali*, James E. Kirby* and George A. O'Doherty*

Department of Chemistry, Northeastern University, Boston, MA 02115, USA;

Department of Pathology Beth Israel Deaconess Medical Center, Boston, MA 02115, USA;

Harvard Medical School, Boston MA 02115, USA;

ykang3@bidmc.harvard.edu (Y-S.K.); mone_sii@yahoo.com.br (S.C.S.); kpsmith2201@gmail.com (K.P.S); ksumida16@gmail.com (K.S.); wang.yuhan8@northeastern.edu (Y.W.)

* Correspondence: lchiarav@bidmc.harvard.edu (L.C); r.donthiri@northeastern.edu (R.R.D.); aljahdali.al@northeastern.edu (A.Z.A.); jekirby@bidmc.harvard.edu (J.E.K.); g.odoherty@neu.edu (G.A.O.)

† Co-first authors, the order is alphabetical.

Table of Contents

1H and 13C NMR Spectra of **2a**	S_2
1H and 13C NMR Spectra of **2b**	S_3
1H and 13C NMR Spectra of **2c**	S_4
1H and 13C NMR Spectra of **3a**	S_5
1H and 13C NMR Spectra of **3b**	S_6
1H and 13C NMR Spectra of **4a**	S_7
1H and 13C NMR Spectra of **4b**	S_8
1H and 13C NMR Spectra of **5a**	S_9
1H and 13C NMR Spectra of **5b**	S_{10}
1H and 13C NMR Spectra of **5c**	S_{11}
1H and 13C NMR Spectra of **5d**	S_{12}
1H and 13C NMR Spectra of **5e**	S_{13}
1H and 13C NMR Spectra of **5f**	S_{14}

2a

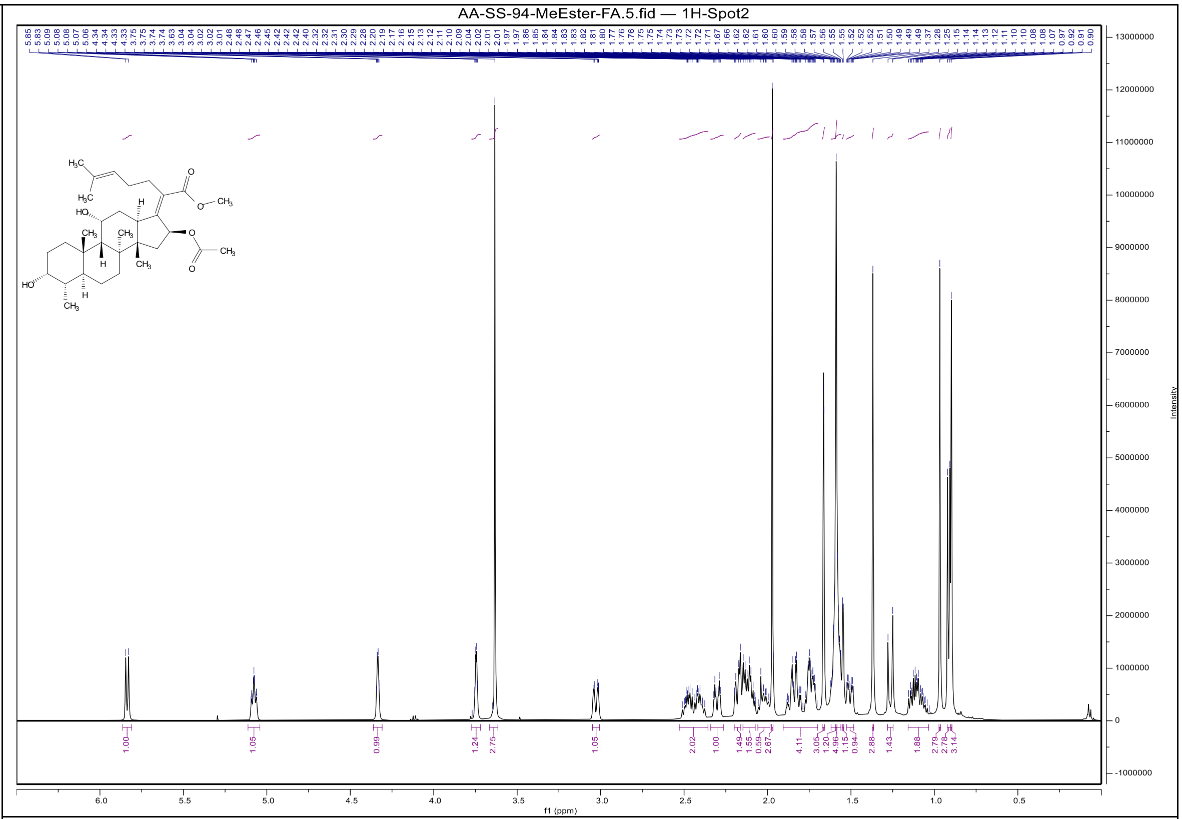

AA-SS-94-MeEster-FA.5.fid — 1H-Spot2
f1 (ppm)
Intensity

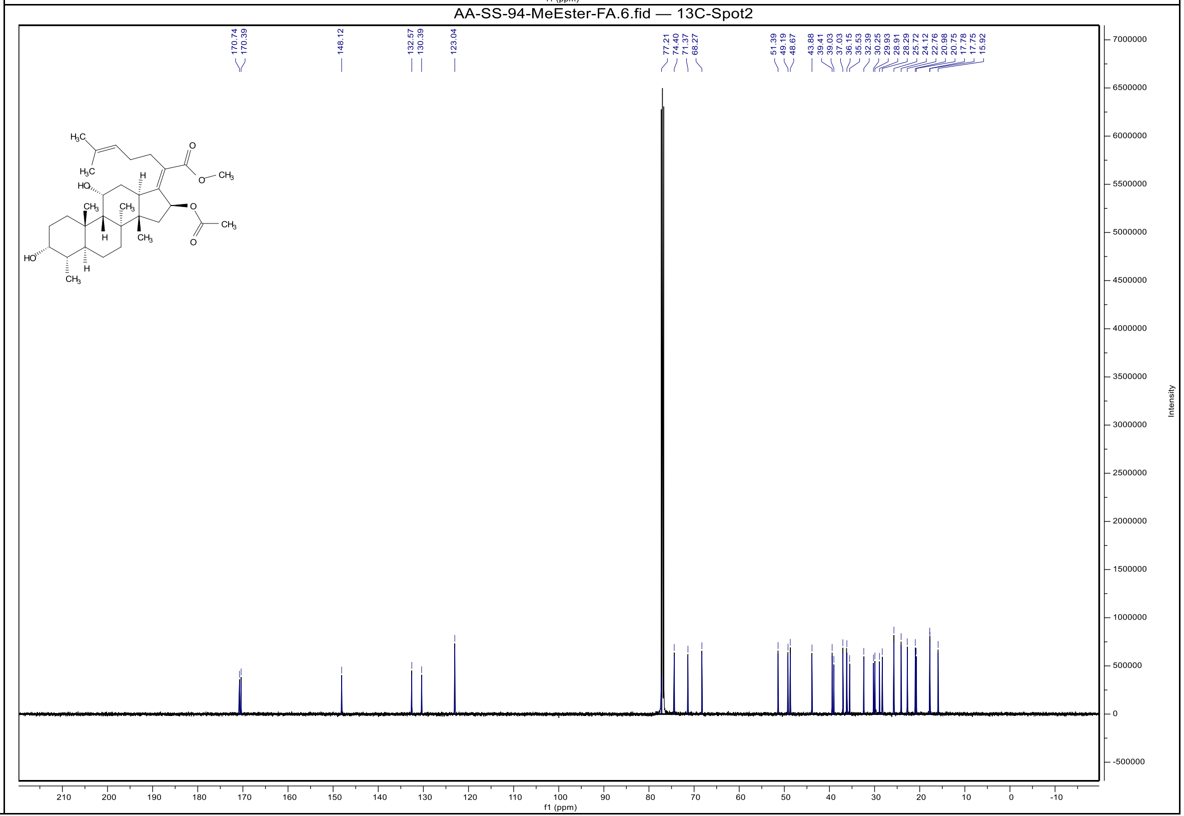

AA-SS-94-MeEster-FA.6.fid — 13C-Spot2
f1 (ppm)
Intensity

2b

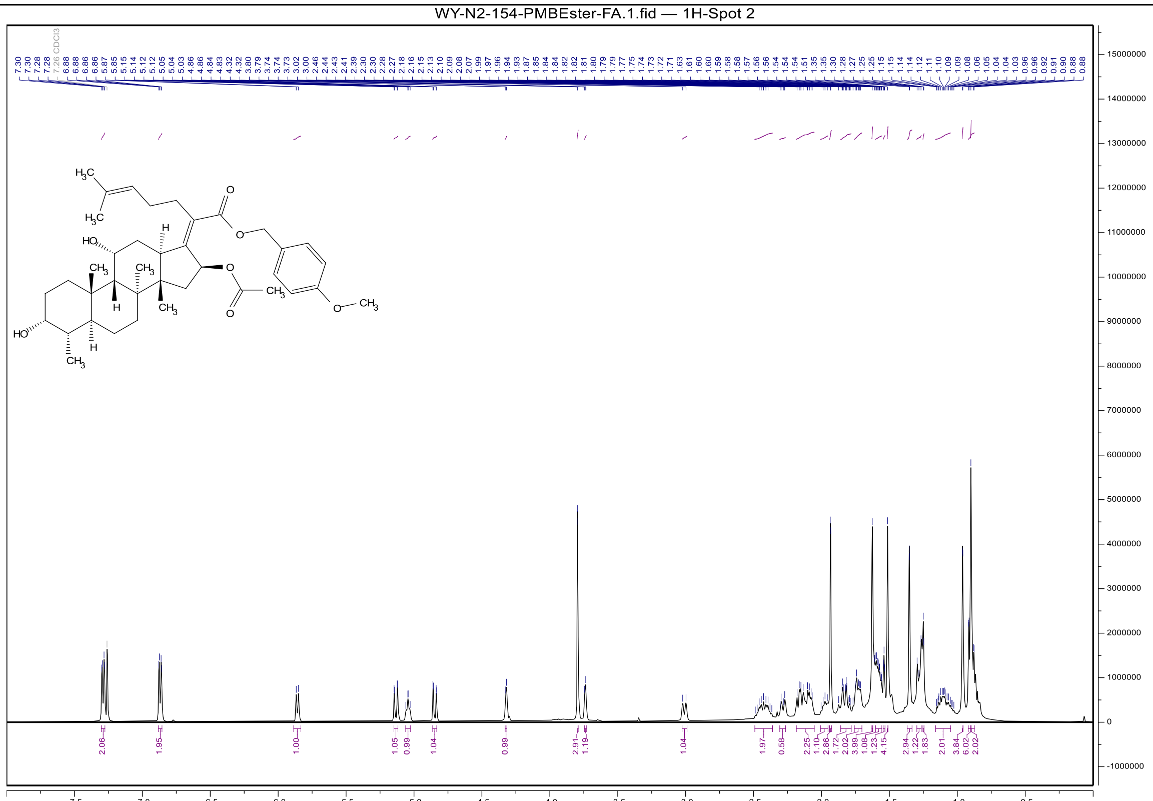
WY-N2-154-PMBEster-FA.1.fid — 1H-Spot 2
f1 (ppm)
Intensity

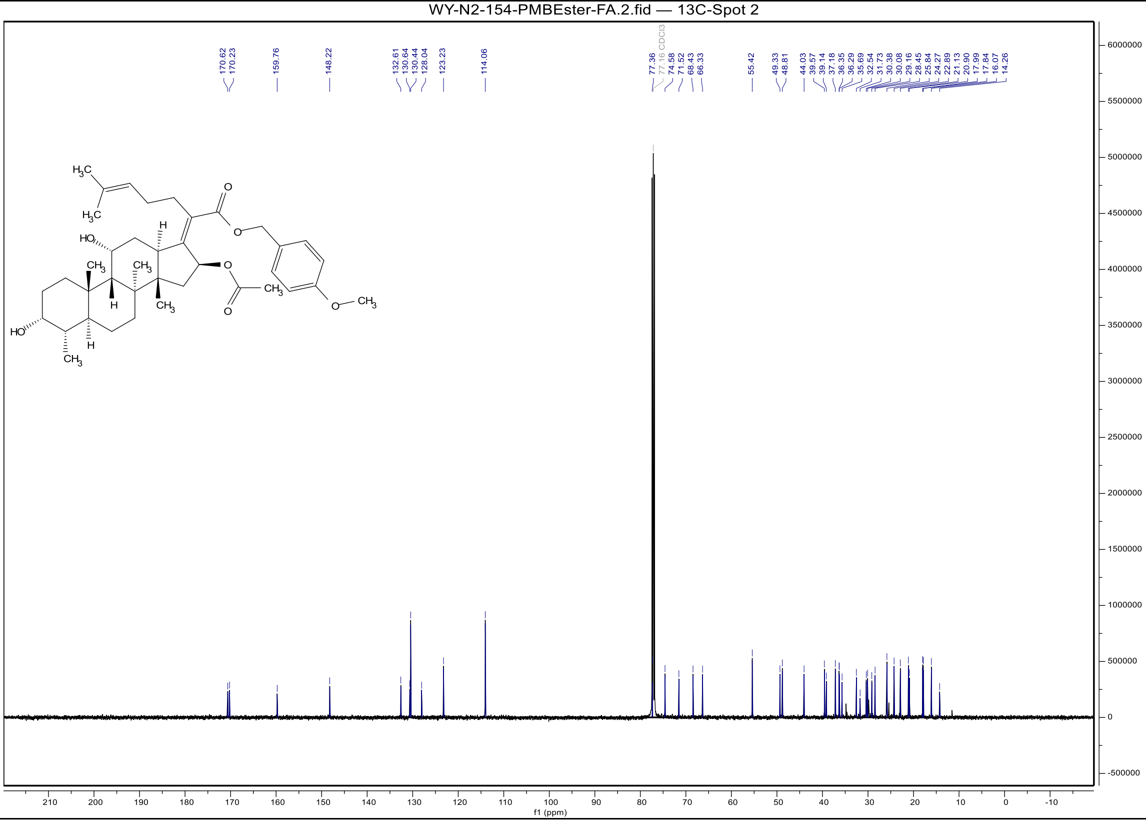
WY-N2-154-PMBEster-FA.2.fid — 13C-Spot 2
f1 (ppm)
Intensity

2c

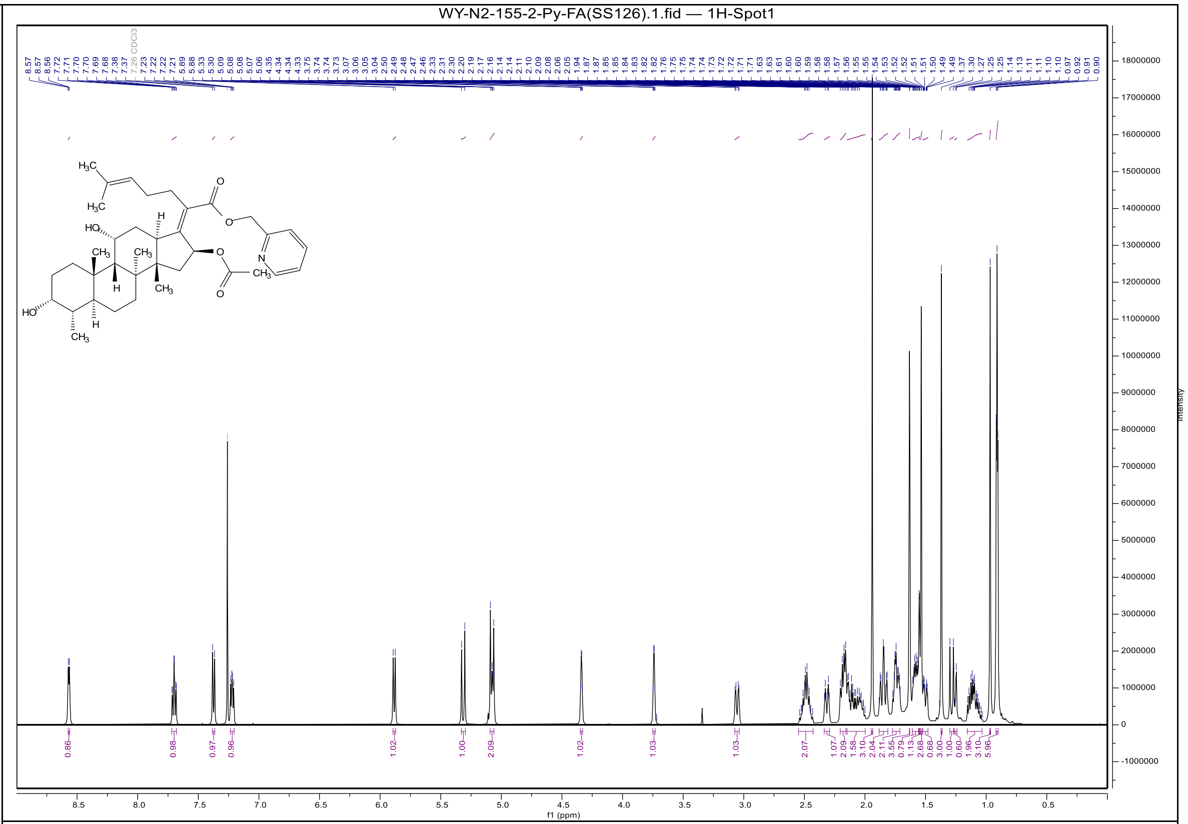
WY-N2-155-2-Py-FA(SS126).1.fid — 1H-Spot1
f1 (ppm)
Intensity

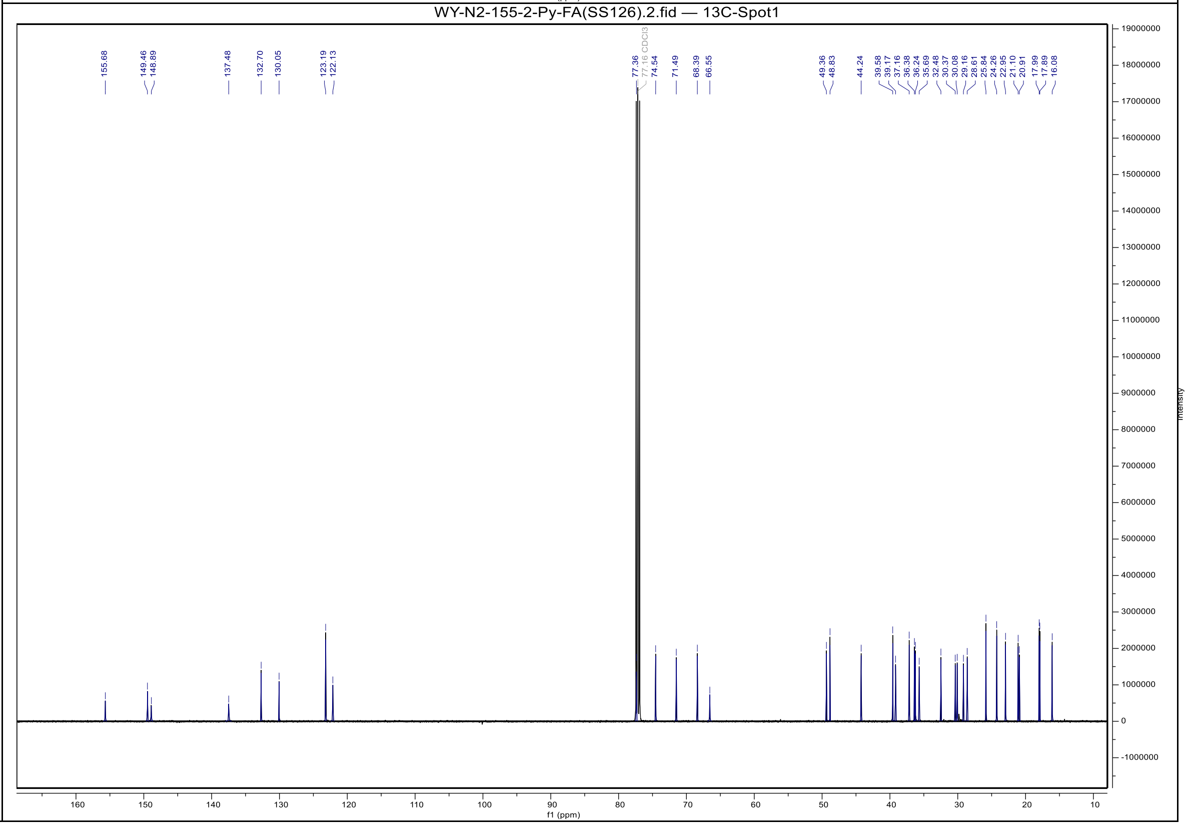
WY-N2-155-2-Py-FA(SS126).2.fid — 13C-Spot1
155.68
149.46
148.89
137.48
132.70
130.05
123.19
122.13
77.36
74.54
71.49
68.39
66.55
49.36
48.83
44.24
39.58
39.17
37.16
36.38
36.24
35.69
32.48
30.37
30.08
29.16
28.61
25.84
24.26
22.95
21.10
20.91
17.99
17.89
16.08
f1 (ppm)
Intensity

3a

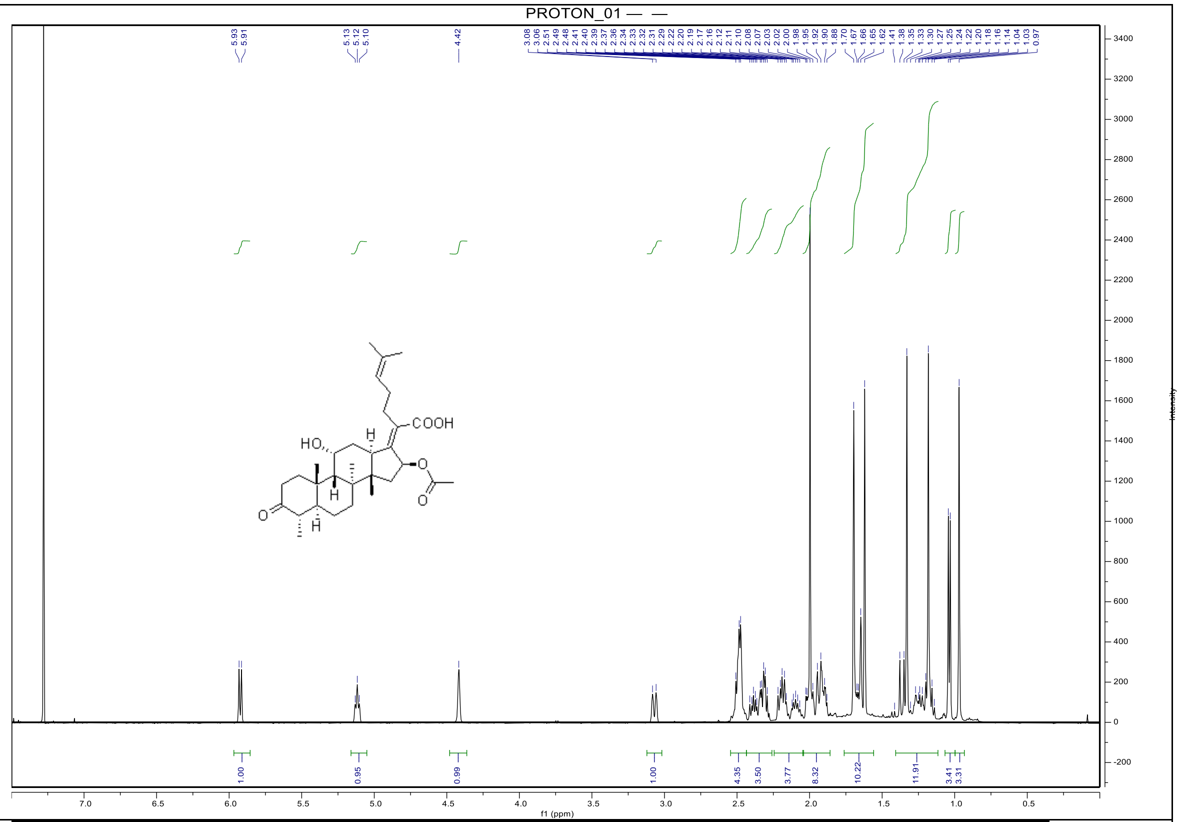

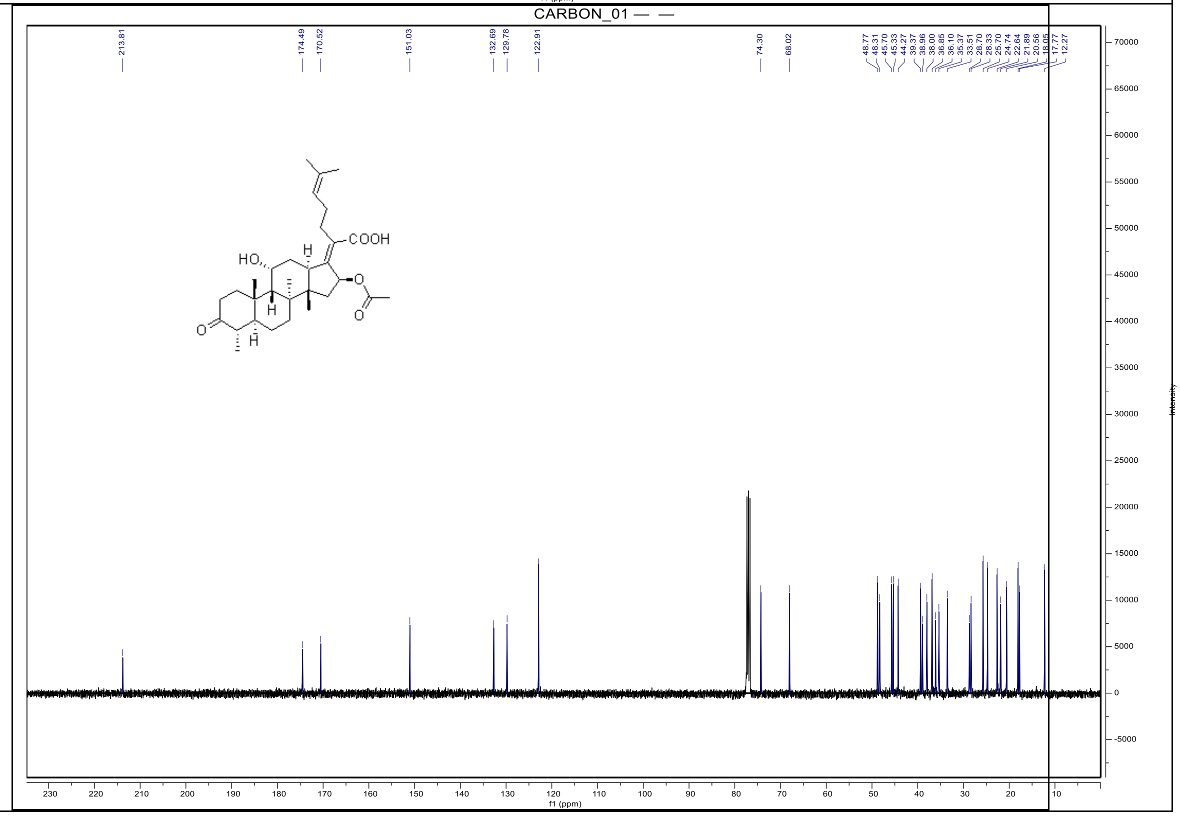

3b

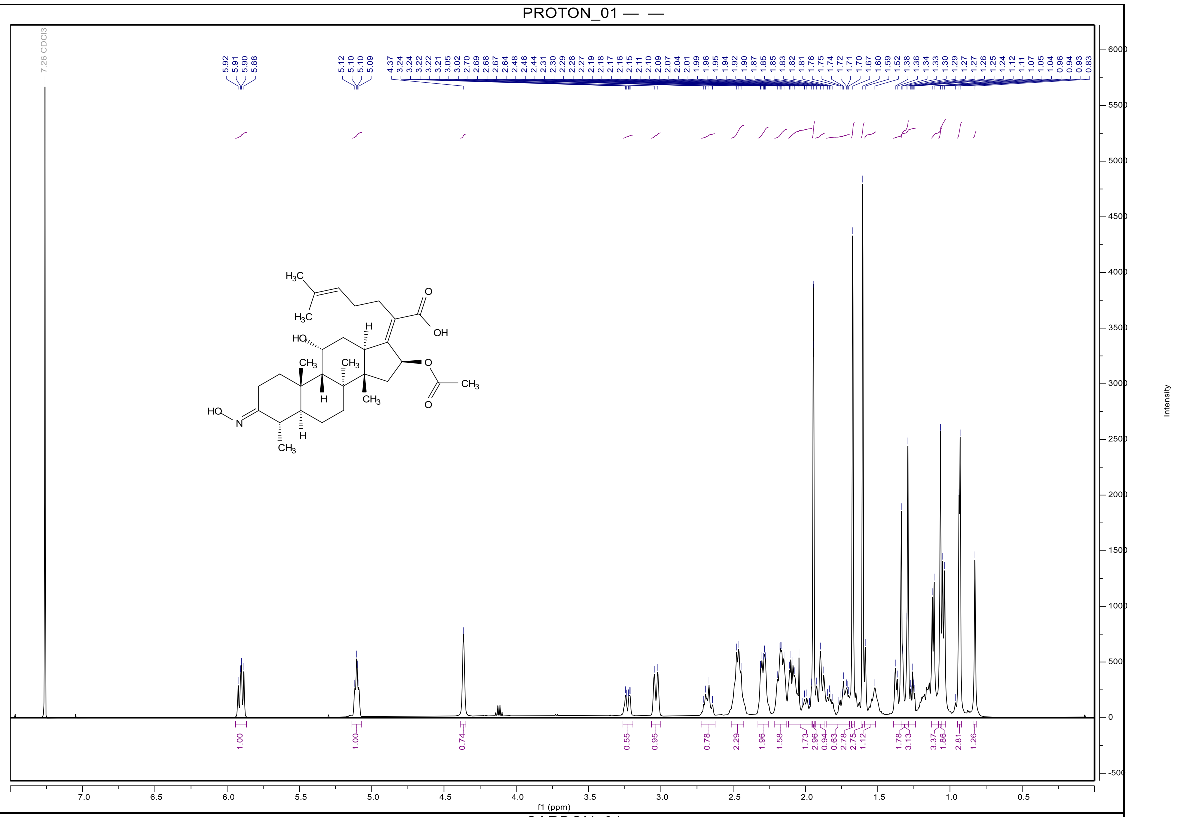

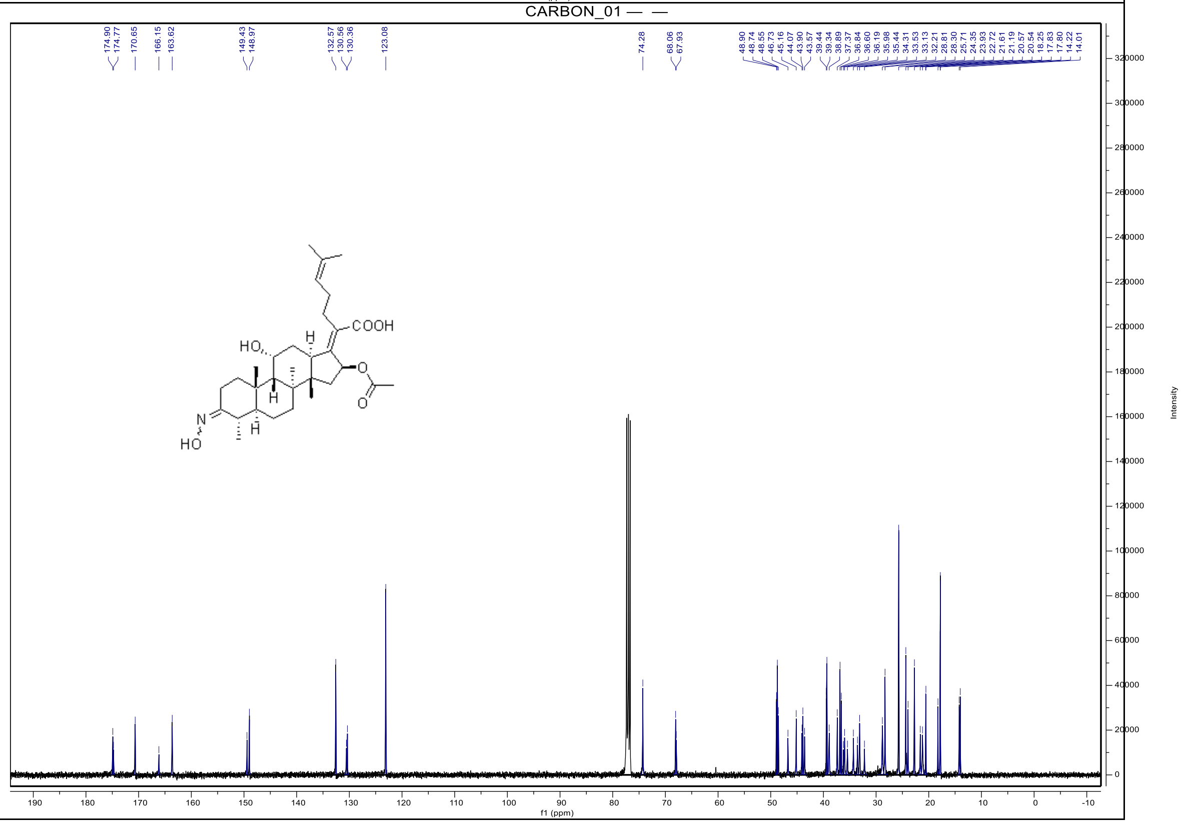

4a

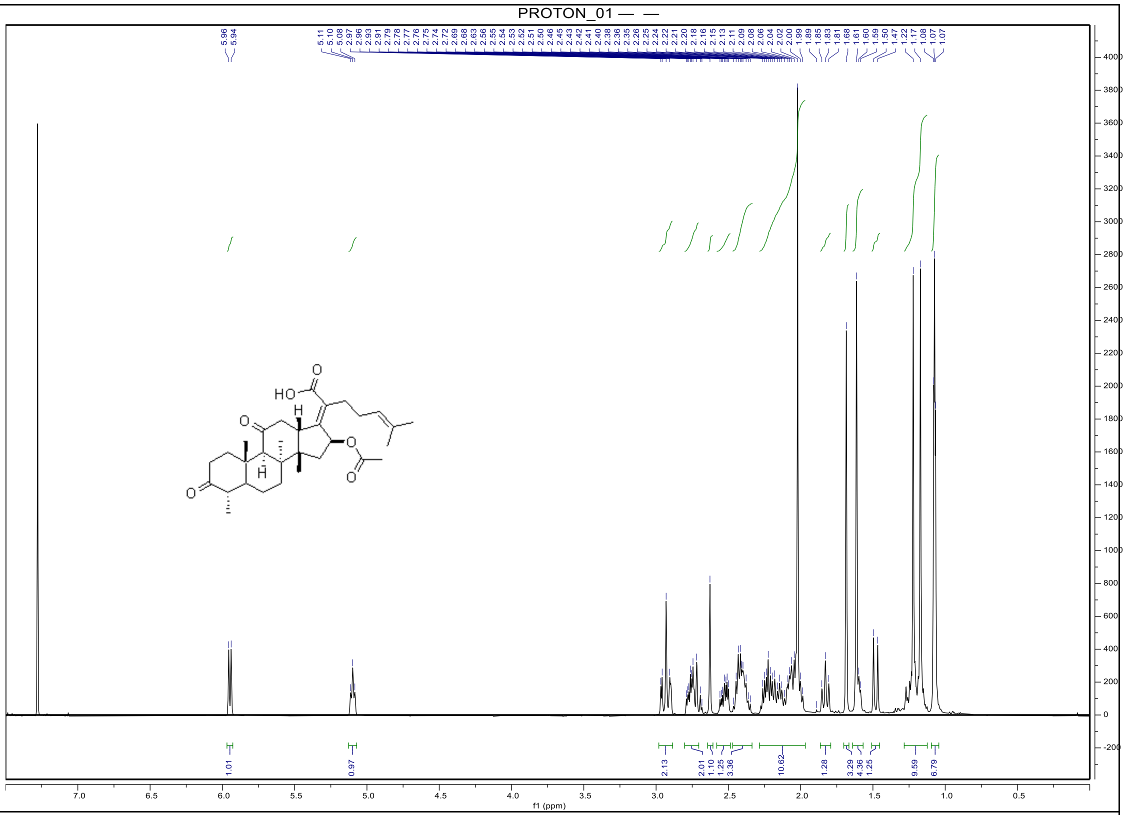
PROTON_01 — —
f1 (ppm)
Intensity

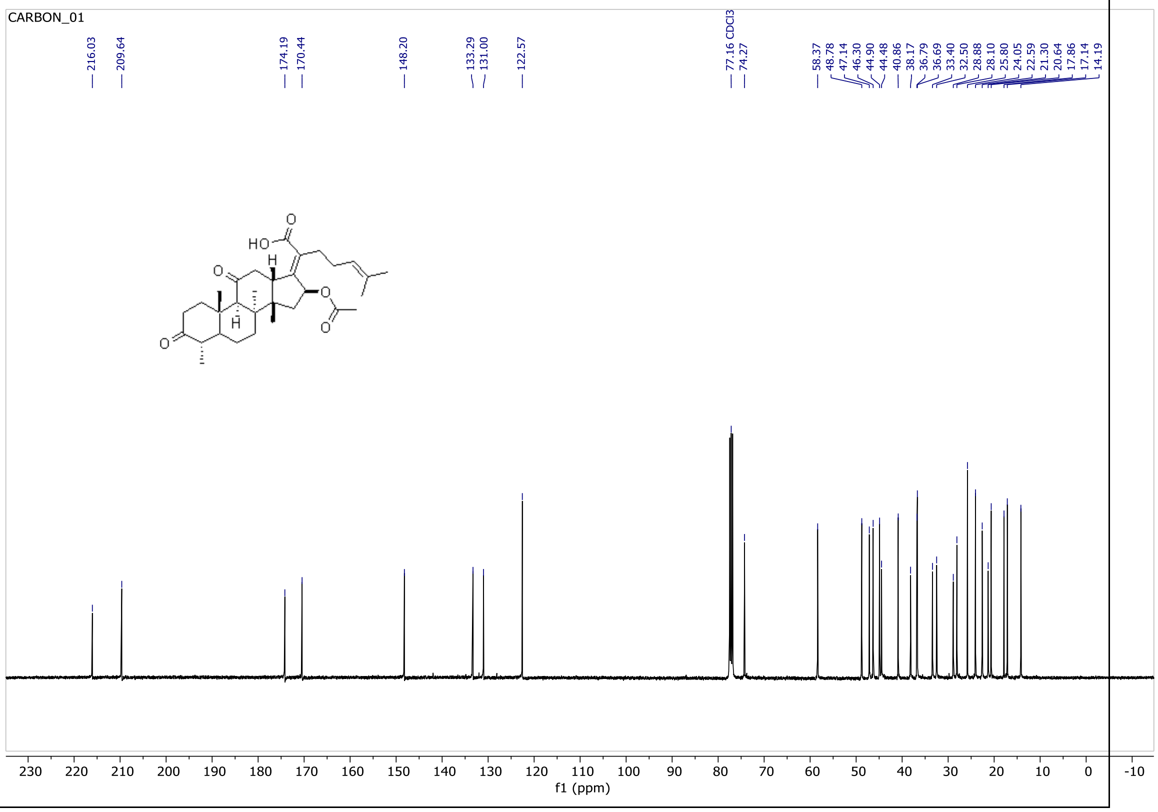
CARBON_01
216.03
209.64
174.19
170.44
148.20
133.29
131.00
122.57
77.16 CDCl3
74.27
58.37
48.78
47.14
46.30
44.90
44.48
40.86
38.17
36.79
36.69
33.40
32.50
28.88
28.10
25.80
24.05
22.59
21.30
20.64
17.86
17.14
14.19
f1 (ppm)

4b

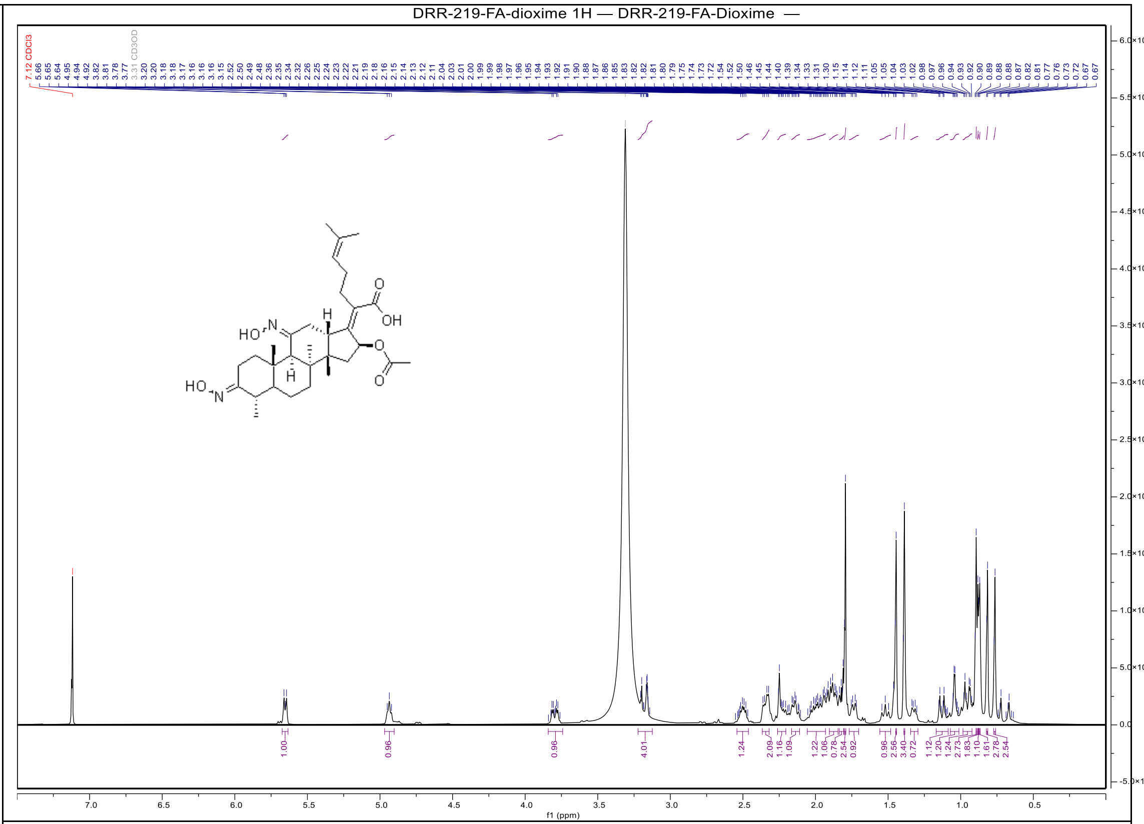
DRR-219-FA-dioxime 1H — DRR-219-FA-Dioxime —
HO
N
HO
N
H
H
O
OH
O
O
f1 (ppm)
Intensity

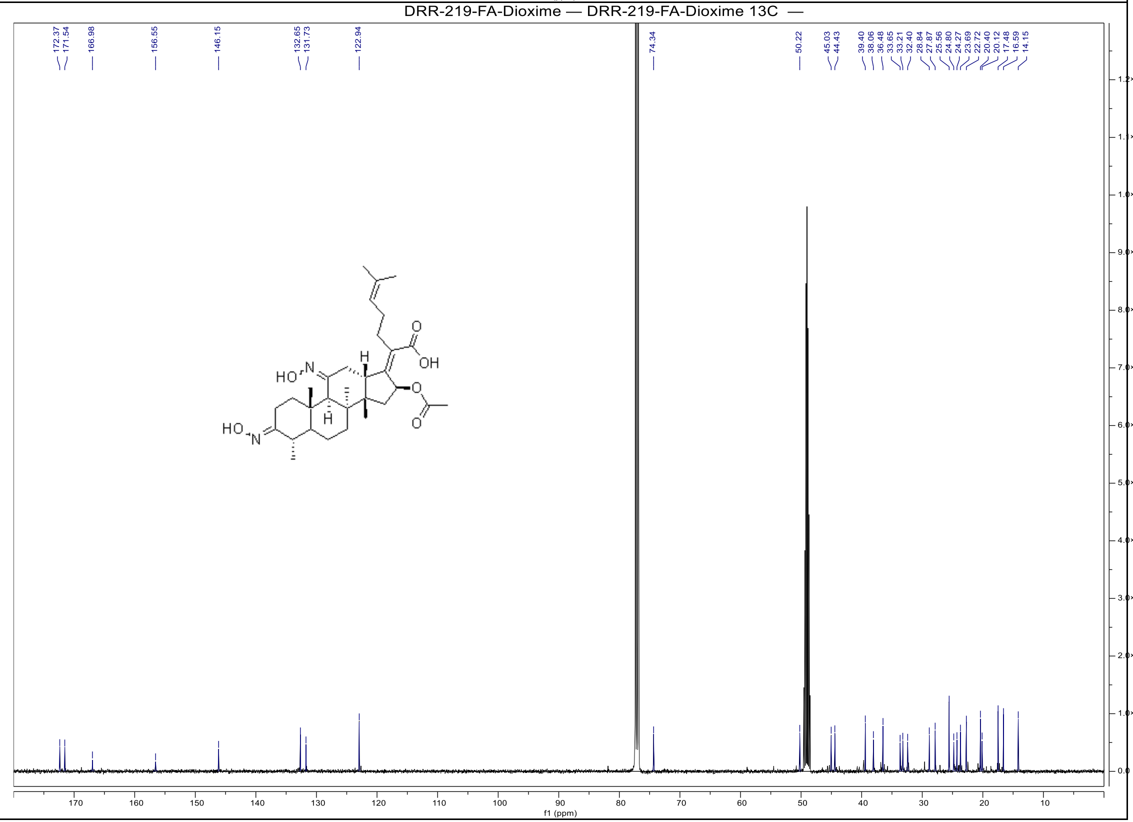
DRR-219-FA-Dioxime — DRR-219-FA-Dioxime 13C —
172.37
171.54
166.98
156.55
146.15
132.65
131.73
122.94
74.34
50.22
45.03
44.43
39.40
38.06
36.48
33.65
33.21
32.40
28.84
27.87
25.56
24.80
24.27
23.69
22.72
20.40
20.12
17.48
16.59
14.15
HO
N
HO
N
H
H
O
OH
O
O
f1 (ppm)
Intensity

5a

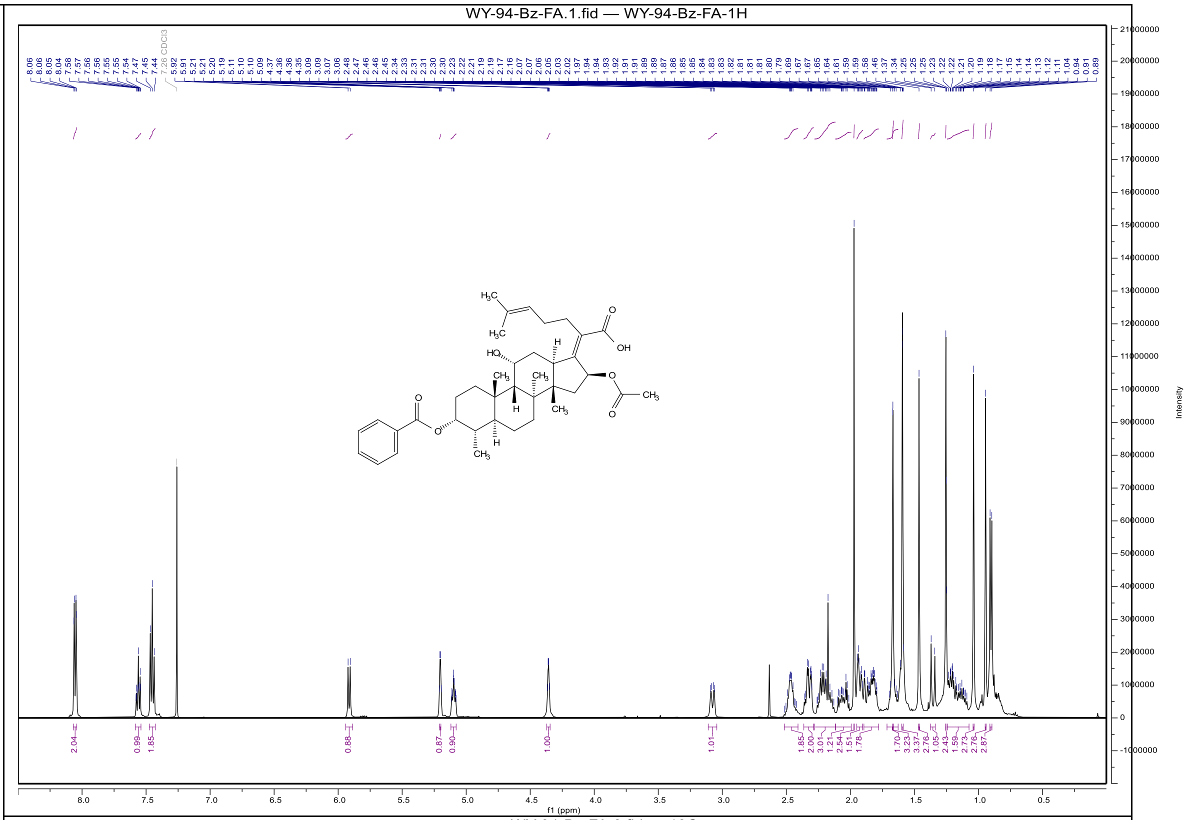
WY-94-Bz-FA.1.fid — WY-94-Bz-FA-1H
f1 (ppm)
Intensity

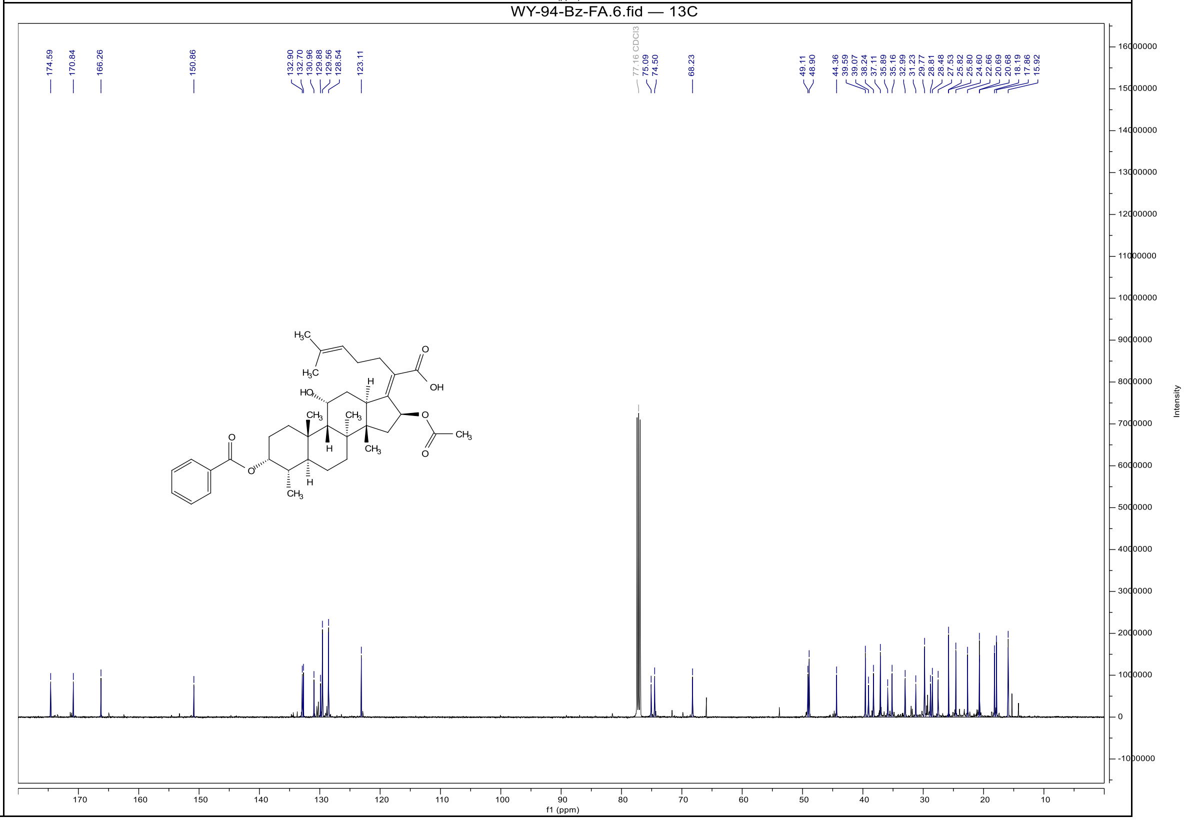
WY-94-Bz-FA.6.fid — 13C
174.59
170.84
166.26
150.86
132.90
132.70
130.96
129.88
129.56
128.54
123.11
77.16 CDCl3
75.09
74.50
68.23
49.11
48.90
44.36
39.59
39.07
38.24
37.11
35.89
35.16
32.99
31.23
29.77
28.81
28.48
27.53
25.82
25.80
24.60
22.66
20.69
20.68
18.19
17.86
15.92
f1 (ppm)
Intensity

5b

WY-92-Picolinic-FA.1.fid — WY-92-Picolinic-FA-1H (Nov. 21, 2023)

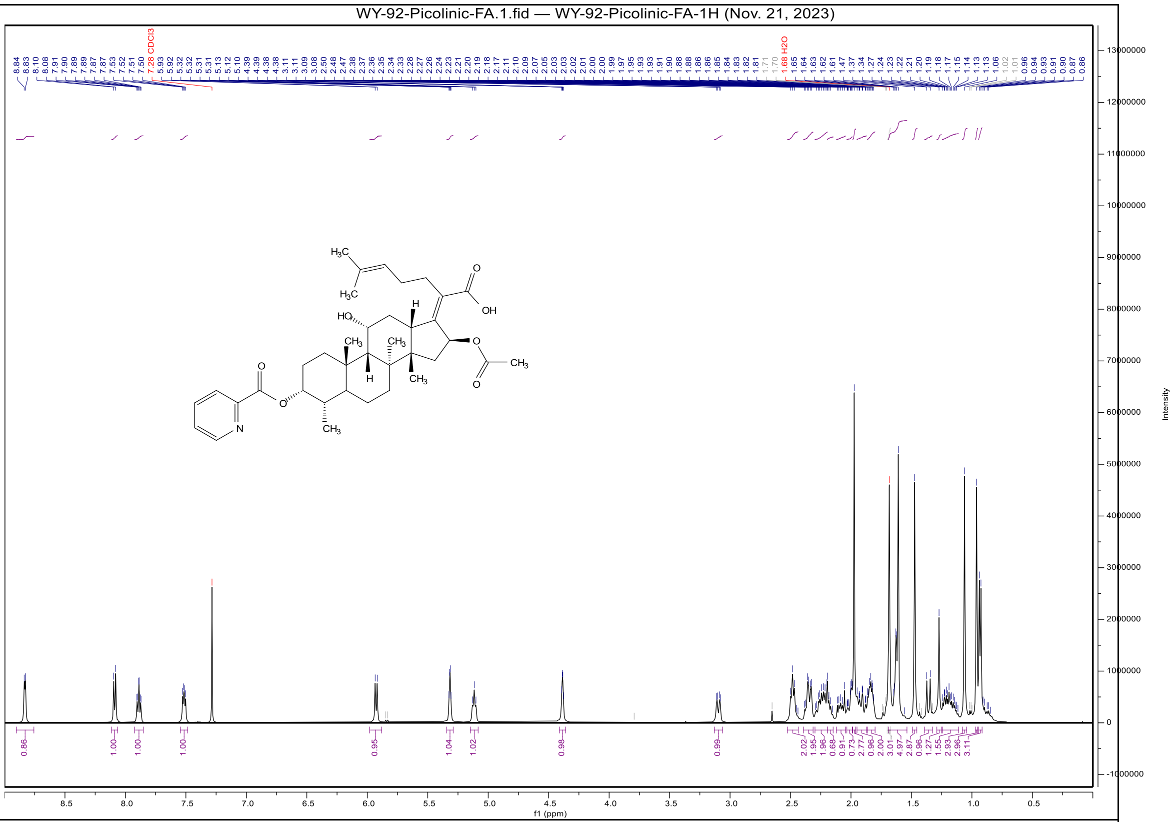

WY-92-Picolinic-FA.2.fid — WY-92-Picolinic-FA-13C

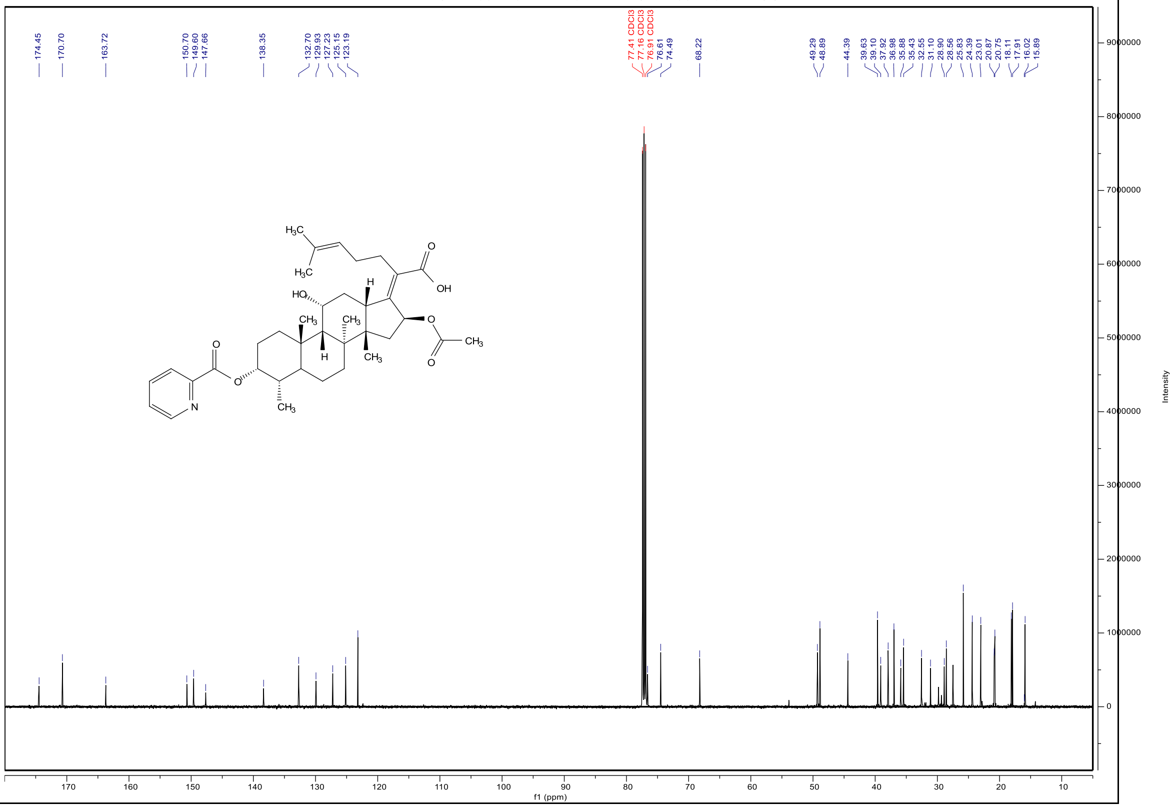

5c

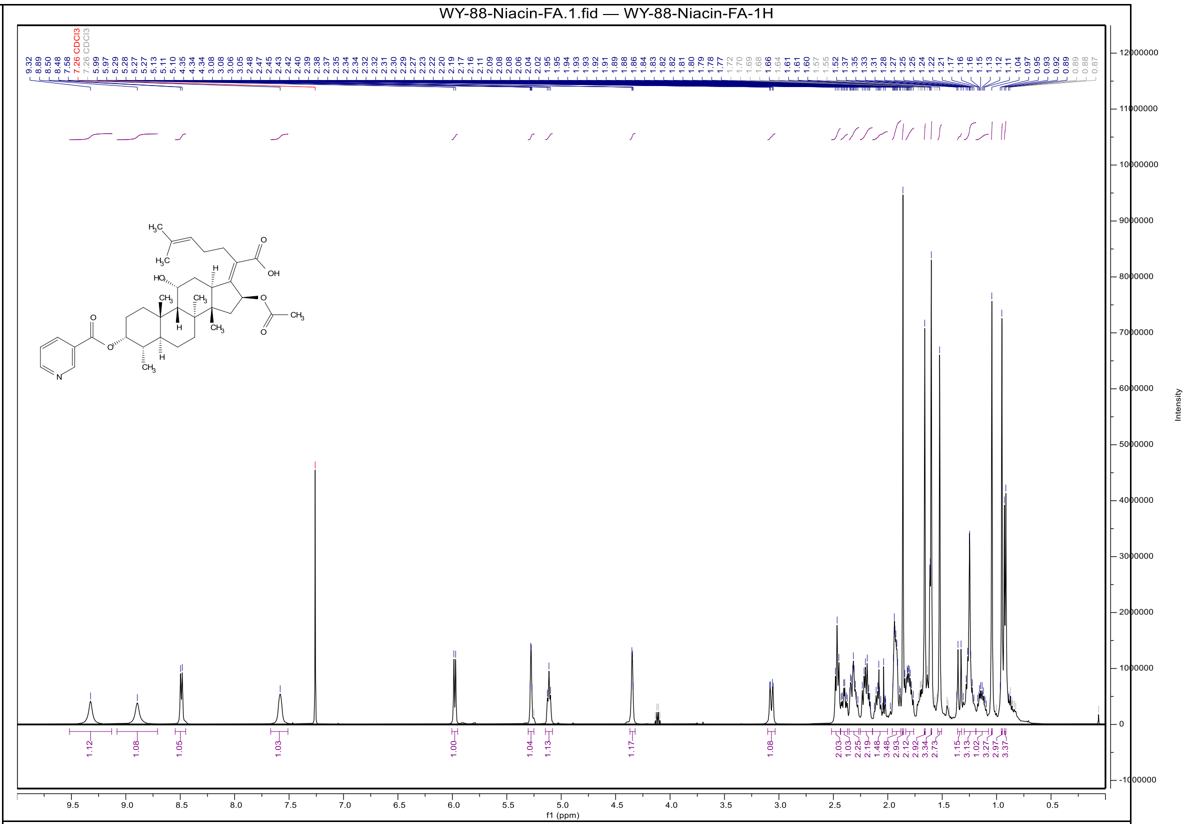

WY-88-Niacin-FA.1.fid — WY-88-Niacin-FA-1H
f1 (ppm)
Intensity

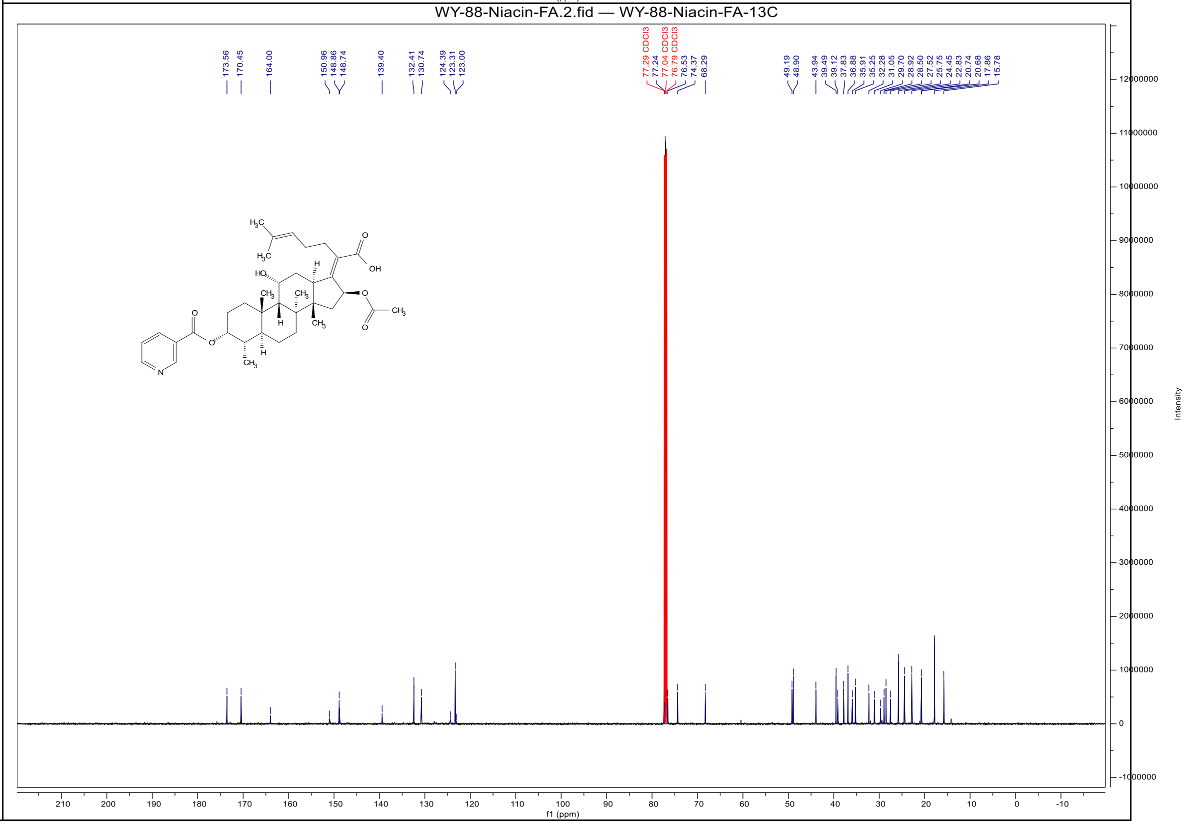

WY-88-Niacin-FA.2.fid — WY-88-Niacin-FA-13C
f1 (ppm)
Intensity

5d

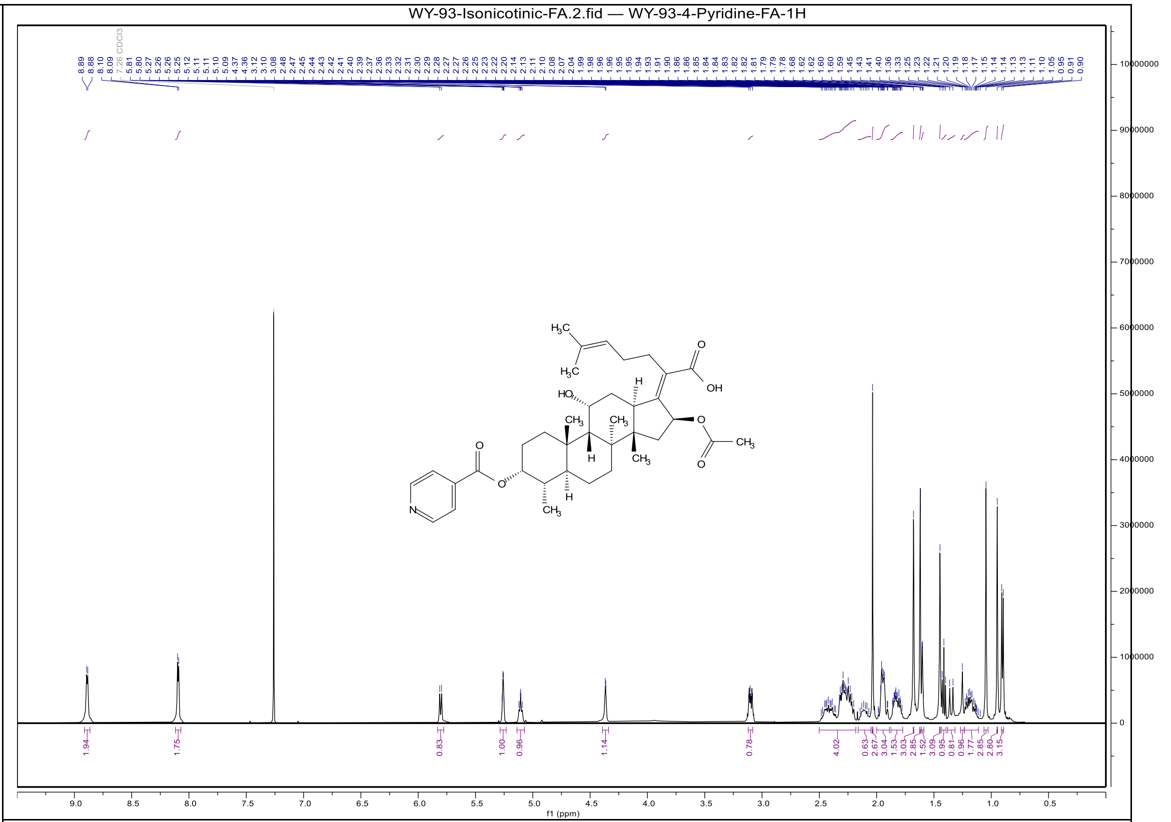
WY-93-Isonicotinic-FA.2.fid — WY-93-4-Pyridine-FA-1H
f1 (ppm)
Intensity

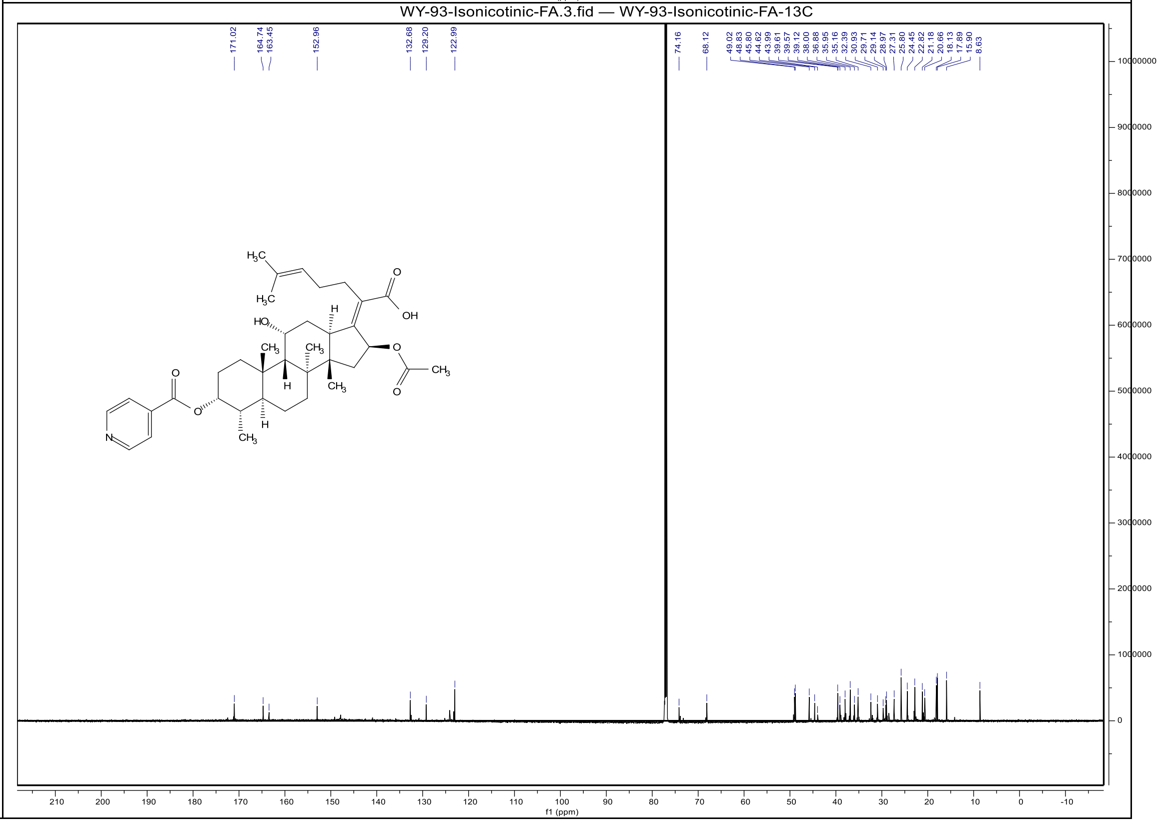
WY-93-Isonicotinic-FA.3.fid — WY-93-Isonicotinic-FA-13C
171.02
164.74
163.45
152.96
132.68
129.20
122.99
74.16
68.12
f1 (ppm)
Intensity

5e

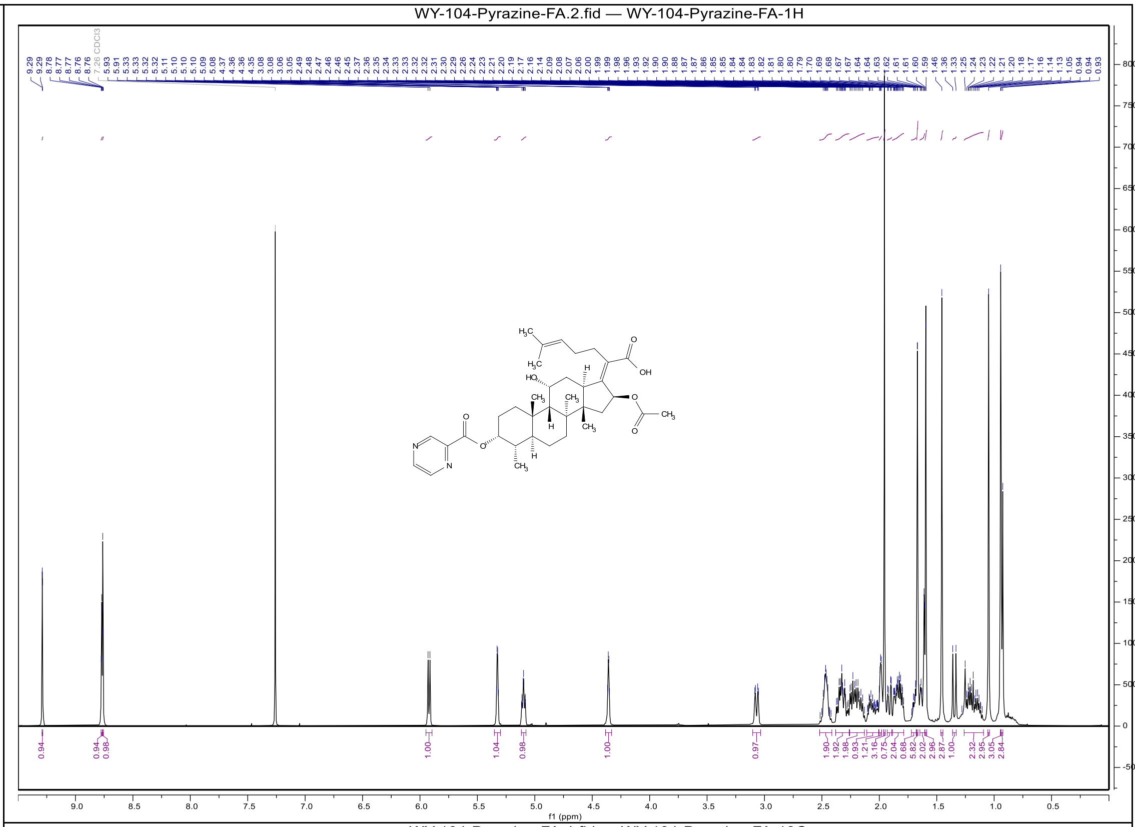
WY-104-Pyrazine-FA.2.fid — WY-104-Pyrazine-FA-1H
f1 (ppm)
Intensity

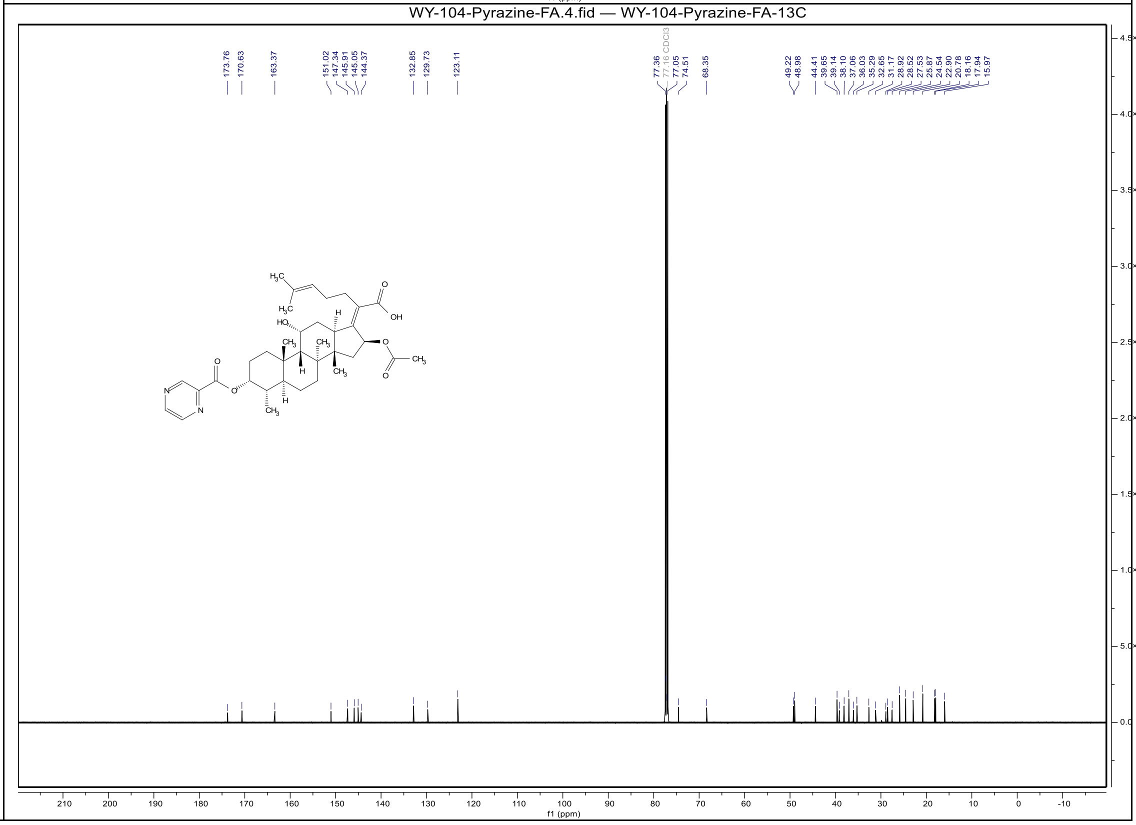
WY-104-Pyrazine-FA.4.fid — WY-104-Pyrazine-FA-13C
173.76
170.63
163.37
151.02
147.34
145.91
145.05
144.37
132.85
129.73
123.11
77.36
77.05
74.51
68.35
49.22
48.98
44.41
39.65
39.14
38.10
37.06
36.03
35.29
32.65
31.17
28.92
28.52
27.53
25.87
24.54
22.90
20.78
18.16
17.94
15.97
f1 (ppm)
Intensity

5f

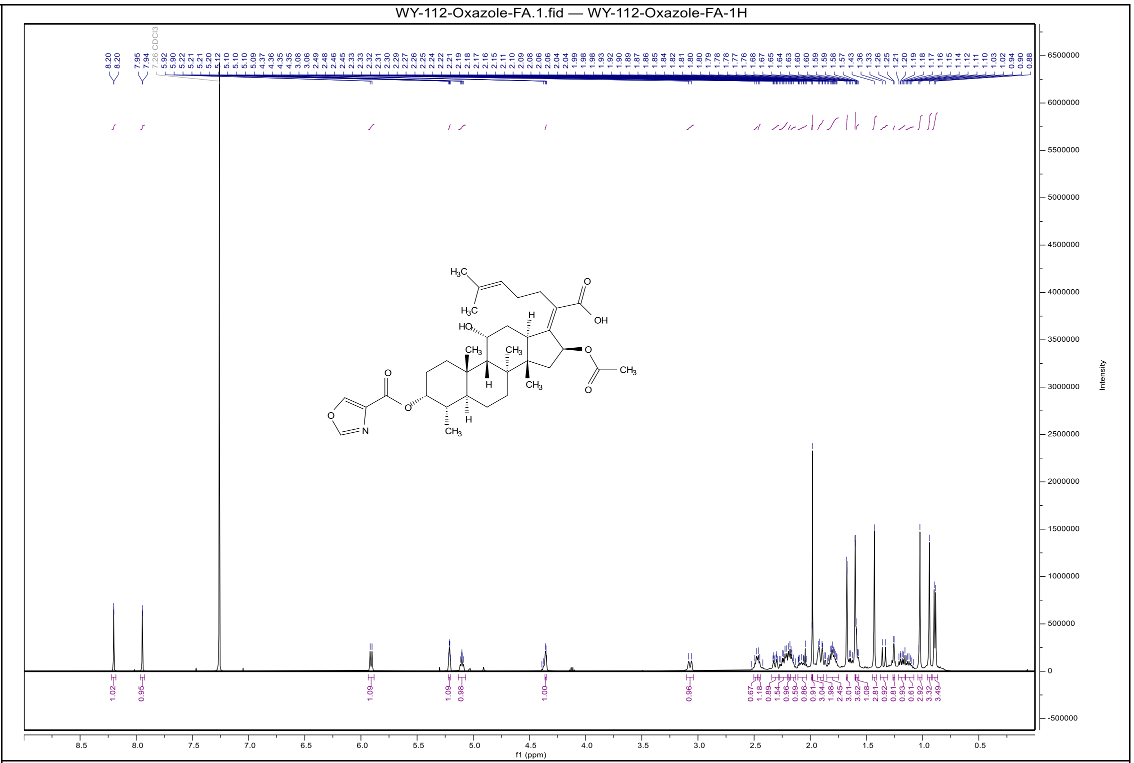
WY-112-Oxazole-FA.1.fid — WY-112-Oxazole-FA-1H
f1 (ppm)
Intensity

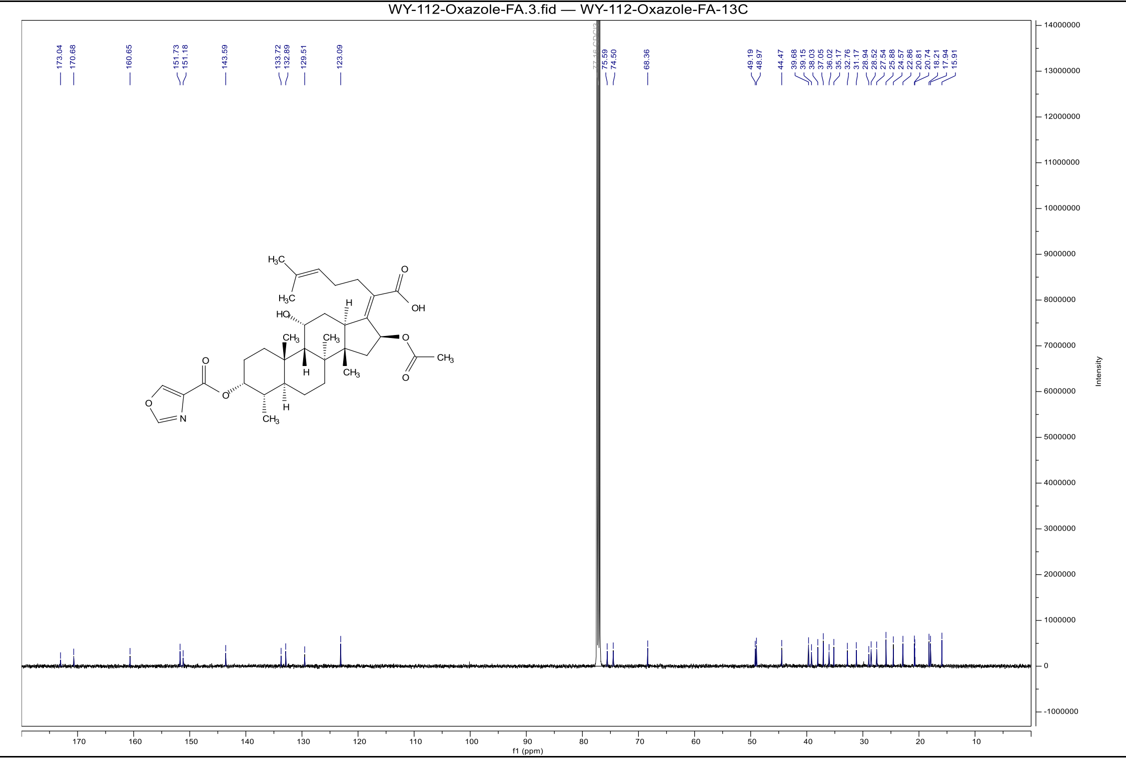
WY-112-Oxazole-FA.3.fid — WY-112-Oxazole-FA-13C
173.04
170.68
160.65
151.73
151.18
143.59
133.72
132.89
129.51
123.09
75.59
74.50
68.36
49.19
48.97
44.47
39.68
39.15
38.03
37.05
36.02
35.17
32.76
31.17
28.94
28.52
27.54
25.58
24.57
22.86
20.81
20.74
18.21
17.94
15.91
f1 (ppm)
Intensity
